# HLA‐B Serine 116 Confers Protection Against Severe COVID‐19 in a Cohort From Rio de Janeiro, Brazil

**DOI:** 10.1111/tan.70479

**Published:** 2025-11-28

**Authors:** Maria Aparecida da Silva Pereira, Sâmila Natiane Ferreira, Cássia Cristina Alves Gonçalves, Mirella Carneiro dos Reis, Carolina da Paz Zampier, Isabela de Carvalho Leitão, Monique de Souza Almeida Lopes, Artur Duque Rossi, Sheila Coelho Soares‐Lima, Rafael Mello Galliez, Shana Priscila Coutinho Barroso, Orlando da Costa Ferreira Junior, Amilcar Tanuri, Terezinha Marta Pereira Pinto Castiñeiras, Átila Duque Rossi, Cynthia Chester Cardoso

**Affiliations:** ^1^ Laboratório de Virologia Molecular, Departamento de Genética, Instituto de Biologia Universidade Federal Do Rio de Janeiro Rio de Janeiro Brazil; ^2^ Laboratório de Biologia Molecular e Proteômica Do Sangue, Instituto de Química Universidade Federal do Rio de Janeiro Rio de Janeiro Brazil; ^3^ Núcleo de Enfrentamento e Estudos de Doenças Infecciosas Emergentes e Reemergentes Universidade Federal do Rio de Janeiro Rio de Janeiro Brazil; ^4^ Clínica de Doenças Infecto Parasitárias Hospital Naval Marcílio Dias Rio de Janeiro Brazil; ^5^ Laboratório de Biologia Molecular, Instituto de Pesquisas Biomédicas Hospital Naval Marcílio Dias Rio de Janeiro Brazil; ^6^ Laboratório de Epigenômica Em Oncologia, Divisão de Pesquisa Clínica e Desenvolvimento Tecnológico Instituto Nacional de Câncer Rio de Janeiro Brazil; ^7^ Programa de Carcinogênese Molecular Instituto Nacional de Câncer Rio de Janeiro Brazil; ^8^ Laboratório de Modelagem e Dinâmica Molecular, Instituto de Biofísica Carlos Chagas Filho Universidade Federal Do Rio de Janeiro Rio de Janeiro Brazil

**Keywords:** COVID‐19, HLA, polymorphism, severity

## Abstract

COVID‐19 is a respiratory disease caused by SARS‐CoV‐2, in which severe outcomes are primarily driven by an exacerbated immune response. The HLA region has been extensively investigated in COVID‐19 due to its central role in the immune response, although genetic associations vary across populations. Here, the association of HLA genetic variability with COVID‐19 severe respiratory outcomes was investigated in an admixed population from Rio de Janeiro, Brazil. Results of a comparative study between mild and severe COVID‐19 cases involving 306 individuals have suggested risk associations with severe COVID‐19 for the HLA‐DPB1*13:01 allele (OR = 3.42, 95% CI = 1.05–11.16, *p* = 0.041) and the HLA‐B*39 allele group (OR = 3.26, 95% CI = 1.16–9.13, *p* = 0.024), although statistical significance was lost after FDR adjustment for multiple comparisons (adjusted *p* > 0.05). Amino acid analyses showed that a serine at position 116 of HLA‐B conferred protection against severe COVID‐19 (OR = 0.4774, 95% CI = 0.28–0.81, *p* = 0.006, adjusted *p* = 0.031). In silico analysis using the NetMHCpan tool predicted that this residue, located in the HLA‐B peptide‐binding groove, has enhanced binding affinity to immunodominant SARS‐CoV‐2 epitopes, suggesting a functional mechanism underlying the observed protection. The association of single nucleotide variants at the HLA region was also investigated, and no statistically significant association was found. Results obtained in the present study underscore the importance of HLA in COVID‐19 severity, likely mediated by its influence on viral peptide presentation, and advance our understanding of the genetic underpinnings of severe disease in admixed populations.

## 1 Introduction

The disease caused by SARS‐CoV‐2, known as COVID‐19, presents a wide range of clinical manifestations, from asymptomatic to severe cases, which may develop critical respiratory outcomes and multiple organ failure, potentially leading to death [1]. In Brazil, the mortality rate reached 1.9% by late 2023, positioning the country as having the fifth highest mortality rate worldwide [2].

Demographic and clinical risk factors, such as advanced age and pre‐existing clinical conditions were the first to be identified as risk factors for severe disease at the beginning of the pandemic [3]. In addition to these factors, numerous studies worldwide have demonstrated the importance of the host's genetic background in disease severity, using both genome‐wide and candidate‐gene approaches. Genes related to viral entry, airway mucus defense, and Type I interferon (IFN‐β) pathways have been consistently associated with disease severity [4, 5, 6]. Indeed, an exacerbated immune response, including a cytokine storm, is commonly observed in severe cases [7], highlighting a key role for genes involved in immunoinflammatory pathways.

The HLA genes, located in the 6p21 chromosomal region, are the most polymorphic region of the human genome [8]. Due to their essential function in regulating immune responses, the HLA complex has been extensively studied in the context of infectious diseases, including COVID‐19. Notably, the binding affinity between HLA‐peptide–T cell receptor complexes influences both humoral and cellular immune responses and plays a critical role in determining COVID‐19 outcomes [9, 10]. While genome‐wide association studies (GWAS) have shown limited signals within the HLA region [11, 12], candidate gene association studies have highlighted correlations between specific HLA alleles and COVID‐19 severity [13, 14, 15]. However, findings observed across different populations are often conflicting, suggesting potential population‐specific effects and reinforcing the importance of replication studies involving individuals from diverse genetic backgrounds and/or transethnic meta‐analysis studies. Given the limited knowledge about the association between HLA alleles and COVID‐19 outcomes in admixed populations and considering recent evidence showing that the Brazilian population is the most admixed in the world [16], this study investigated the association of HLA alleles with COVID‐19 in a population with a complex genetic background from Rio de Janeiro, Brazil.

## 2 Materials and Methods

### 2.1 Study Participants

A total of 306 individuals aged 18 years or older were enrolled in a case–control study designed to investigate genetic factors associated with progression to severe respiratory outcomes of COVID‐19. Therefore, RT‐qPCR confirmed SARS‐CoV‐2 infection was defined as an inclusion criterion for the study. All subjects were diagnosed between March and December 2020, before the introduction of vaccines and the emergence of variants of concern. Patients who required hospitalisation and showed intense respiratory distress—defined here by their need for oxygen therapy—were classified as cases (severe disease, *N* = 114). These patients were recruited at Hospital Naval Marcílio Dias (HNMD), a reference hospital located in the municipality of Rio de Janeiro, which offers tertiary care for high‐complexity cases of several conditions, including COVID‐19 cases. The comparison group included 192 individuals with mild COVID‐19 symptoms throughout the entire disease course, recruited at the Núcleo de Enfrentamento e Estudos de Doenças Infecciosas Emergentes e Reemergentes from Universidade Federal do Rio de Janeiro (NEEDIER/UFRJ) a centre for molecular diagnostic and clinical follow‐up of mild COVID‐19 cases also located in Rio de Janeiro. Thus, all study participants were recruited from the same geographic area. Clinical and demographic data were obtained either from medical records (severe cases) or through a questionnaire administered by a qualified medical specialist (mild COVID‐19 cases). The groups were matched by sex, and by the presence of at least one of the main comorbidities associated with COVID‐19 severity (hypertension and/or diabetes). The study was approved by the Brazilian National Commission of Ethics in Research and by the institutional ethical review board from HNMD (protocol numbers 30161620.0.0000.5257 and 32382820.3.0000.5256, respectively) and written informed consent was obtained from all participants.

### 2.2 Sample Processing and Genotyping

Genomic DNA was extracted from peripheral blood and nasopharyngeal swab samples using the QIAamp DNA Blood Mini Kit (QIAGEN, Germany), following the manufacturer's instructions. Genotyping was performed using the Infinium Global Screening Array‐24 v3.0 (Illumina Inc.), which includes 654,027 markers along the human genome, according to the standard Infinium High‐throughput Screening (HTS) protocol. Signal intensities were acquired using the iScan instrument (Illumina Inc.). Genotypes were determined by GenomeStudio 2.0 software (Illumina Inc.) using the GRCh37 genome reference. PLINK Input Report Plug‐in v2.1.4 was applied to export data in PED and MAP formats.

### 2.3 Quality Control and Ancestry Inference

Genotype data from autosomal chromosomes were extracted and quality control was performed using PLINK 1.9 [17] according to published guidelines [18, 19]. Briefly, single nucleotide polymorphisms (SNPs) with call rates under 95% or minor allele frequency (MAF) below 5% were excluded. Deviations from Hardy–Weinberg equilibrium were assessed by chi‐squared tests (*α* = 10^−6^) using the dataset from the mild cases group and SNPs showing deviations were also excluded. The dataset obtained after SNPs quality control was used to determine sample call rates, and those below 80% were removed from further analysis. The threshold applied for sample call rates (80%), although more permissive than the usual 90%–95%, is adequate to ensure sufficient data quality while minimising the loss of statistical power given the reduced sample size [19].

Data from the mild COVID‐19 group was also used to determine linkage disequilibrium (LD) patterns, and the complete dataset was pruned using an *r* ^2^ > 0.2 as a cutoff.

Ancestry inference was performed using samples from the 1000 Genomes Project as reference. The reference panel included individuals from European (EUR) (*N* = 206), African (AFR) (*N* = 207) and Native American (NAM) (*N* = 49) ancestries. Genotype data were retrieved from https://www.internationalgenome.org/data. After filtering the reference panel to match the variants in the pruned study set, the two sources were merged into a final dataset encompassing 767 individuals and 79,963 variants. Proportions of EUR, AFR and NAM ancestries were estimated for each sample through an unsupervised model implemented in the ADMIXTURE software (version 1.3.0) [20].

### 2.4 HLA Genotypes Imputation

Classical HLA Class I (*HLA‐A*, *HLA‐B*, and *HLA‐C*) and HLA Class II (*HLA‐DRB1*, *HLA‐DQA1*, *HLA‐DQB1 HLA‐DPA1*, and *HLA‐DPB1*) alleles and additional variants located at the HLA region were imputed using the Michigan Imputation Server with a multiethnic reference panel [21, 22]. Genotype data from the extended HLA region (chromosome 6: from 25 to 35 Mb, according to GRCh37) were extracted from the primary dataset and formatted according to the Michigan Imputation Server guidelines (https://imputationserver.sph.umich.edu/index.html). Imputation was performed using the Four‐Digit Multi‐Ethnic HLA v2 (2022) reference panel. Only genetic variants and classical alleles with a minimum imputation accuracy (*r* ^2^) of 80% were retained for analysis.

### 2.5 Statistical Analysis

Distribution of age, sex, genetic ancestry and pre‐existing conditions was compared between subjects with severe and mild COVID‐19 using Fisher's exact test and Mann–Whitney test for categorical and continuous variables, respectively. The association of HLA alleles, allele groups and SNPs with COVID‐19 severity was evaluated by logistic regression models adjusted for the covariates age and NAM ancestry, which were selected through a stepwise forward logistic regression [23], as described in Box S1. The association analyses were conducted using four independent approaches, as outlined in Figure S1. First, the association between HLA allelic groups (first‐field) and COVID‐19 severity was assessed by comparing the frequencies of carriers of each allele group between patients with severe and mild disease using logistic regression models adjusted for the selected covariates. Analyses were run on R software (version 4.2.2) and the package “genetics.” Logistic regression models were also fitted to test the association of HLA alleles at two‐fields with severe disease. These analyses were conducted using the MiDAS [24] package in R, under additive and dominant models. The HWE.test function was applied to assess deviations from Hardy–Weinberg equilibrium, with a significance level of 0.05. To avoid biases from extremely rare alleles, a minimum allele frequency of 0.01 was used as a cutoff. Package MiDAS was also applied to investigate the association between amino acid residues in HLA proteins and COVID‐19 severity, considering alleles with frequency > 0.02. Briefly, residues were inferred from sequence alignments in the IPD‐IMGT/HLA database. Then, logistic regression models were fitted and compared using likelihood‐ratio tests to assess the association between individual residues and disease severity. Finally, associations between single nucleotide variants and disease severity were also assessed through logistic regression under an additive model using PLINK 2.0. False discovery rates (FDRs) were used to adjust *p*‐values for multiple comparisons. Odds ratios (ORs) were determined as effect estimates, along with 95% confidence intervals (95% CIs).

### 2.6 HLA Peptide‐Binding Affinity Predictions

The NetMHCpan 4.1a [25] tool was used for in silico prediction of peptide‐binding affinity for selected HLA Class I allotypes, which were previously identified through residue‐level association analyses. All possible 9‐mer peptide fragments derived from the SARS‐CoV‐2 Spike glycoprotein were evaluated for both the original Wuhan strain (UniProtKB: P0DTC2; GenBank: YP_009724390.1) [26] and Omicron BA.2.12.1 variant (PDB: 7XNS_C; GenBank: OQ355080.1) [27] to assess whether the enhanced binding affinity pattern was maintained across Variants of Concern. Peptides with predicted binding percentile rank (%Rank) < 0.5 and half‐maximal inhibitory concentration (IC50 < 50 nM) were classified as strong binders, based on established thresholds for MHC Class I epitope prediction.

## 3 Results

### 3.1 Characteristics of the Study Cohort

The study cohort consisted of 306 individuals infected with SARS‐CoV‐2, including 114 cases who required mechanical ventilation and 192 individuals with mild symptoms. After applying quality control, a kinship analysis was performed, and no related pairs were identified. The median ages were 61.63 years for severe cases and 54.98 years for the group with mild COVID‐19. Distribution of sex and comorbidities (hypertension and/or diabetes) was similar between severe and mild cases. Hypertension was the most frequently reported comorbidity, followed by diabetes, which was more prevalent among individuals with severe symptoms. Genetic ancestry estimates showed higher proportions of European ancestry in the study cohort, followed by African and NAM ancestries. These covariates were investigated as potential confounders and, after stepwise logistic regression analysis (Box S1), age and NAM ancestry were selected for inclusion in all association analyses. Demographic data are shown in Table 1.

**TABLE 1:** Demographic, clinical and genetic ancestry of study participants.

	Severe disease (*N* = 114)	Mild symptoms (*N* = 192)	*p* *
Age in years	61.63 (11.44)	54.89 (9.92)	4.55 × 10^−8^
Sex			0.34
Male	71 (62%)	108 (56%)	
Female	43 (38%)	84 (44%)	
Comorbidities	83 (73%)	139 (72%)	0.99
Hypertension	77 (67%)	118 (61%)	0.32
Diabetes	47 (41%)	53 (28%)	0.02
Ancestry (%)			
European	61 (18.67)	66 (23.36)	0.028
African	26.5 (17.24)	27 (22)	0.374
Native American	12.5 (23.37)	7 (6.77)	4.77 × 10^−5^

*Note:* Results are shown as mean and standard deviation for age and genetic ancestry and as counts (*N*) and frequency (%) for sex and comorbidities.

* *p* values obtained from Fisher's test for sex and comorbidities, and from the Mann–Whitney test for age and ancestry.

### 3.2 HLA Alleles and Amino Acid Residues in Association With COVID‐19 Severity

After applying quality control criteria and extraction of the HLA region (25–35 Mb), the filtered dataset included 300 samples with a genotyping rate of 0.992 and 6202 variants. After imputation, a final dataset comprising 14,519 variants and 191 alleles from 8 HLA Class I and HLA Class II genes (*HLA‐A*, *HLA‐B* and *HLA‐C*, *HLA‐DRB1*, *HLA‐DQA1*, *HLA‐DQB1 HLA‐DPA1* and *HLA‐DPB1*) successfully imputed with high accuracy (*r* ^2^ ≥ 0.8) was obtained. Frequencies of these alleles are provided in Table S1.

Results obtained from the allelic group‐level analysis under a dominant model adjusted for age and genetic ancestry (Tables 2 and S2) suggested increased risk of severe COVID‐19 among carriers of the HLA Class I allele group HLA‐B*39 (OR = 3.26; 95% CI = [1.16–9.13]; *p* = 0.024) and the HLA Class II allele HLA‐DPB1*13 (OR = 3.42, 95% CI = [1.05–11.16]; *p* = 0.041). However, these associations did not remain statistically significant after FDR correction for multiple comparisons (adjusted *p* = 0.672 and 0.861, respectively).

**TABLE 2:** Association between COVID‐19 severity and variations in HLA Class I and HLA Class II genes at allelic group, two‐field alleles and amino acid position.

HLA alleles	Severe disease (*N*, %)	Mild symptoms (*N*, %)	OR [95% CI]	*p* *	Adjusted *p* **
Allelic group					
HLA‐B*39	13 (5.7%)	7 (1.82%)	3.26 [1.16–9.13]	0.024	0.672
HLA‐DPB1*13	9 (3.95%)	5 (1.3%)	3.42 [1.05–11.16]	0.041	0.861
Two‐field allele					
HLA‐ DPB1*13:01	9 (3.95%)	5 (1.3%)	3.43 [1.08–12.05]	0.041	0.967
Amino acid					
HLA‐B Serine 116	36 (15.7%)	90 (23.44%)	0.4774 [0.27–0.80]	0.0061	0.031

*Note:* Results were obtained from logistic regression adjusted for age (years) and proportions of Native American ancestry.

Abbreviations: %, frequency; CI, confidence interval; *N*, number of participants; OR, odds ratio.

* *p* values obtained from logistic regression adjusted for age (continuous) and Native American ancestry.

** FDR adjusted *p* value.

Then, the frequency of two‐fields HLA alleles was investigated in mild and severe COVID‐19 cases. Among HLA Class I genes, *HLA‐B* was the most variable, with 47 alleles imputed and HLA‐B*07:02 as the most frequent (6.7%), followed by *HLA‐A* (33 alleles) and *HLA‐C* (27 alleles), with higher frequencies observed for HLA‐A*02:01 and HLA‐C*04:01 alleles (18.8 and 18.6%, respectively). For HLA Class II, *HLA‐DRB1* exhibited the highest variability (33 alleles), with HLA‐DRB1*07:01 as the most frequent (11.4%), followed by *HLA‐DPB1* (23 alleles; HLA‐DPB1*04:01; 25.1%), *HLA‐DQB1* (14 alleles; HLA‐DQB1*02:01; 22.1%), *HLA‐DQA1* (8 alleles; HLA‐DQA1*05:01; 24.6%) and *HLA‐DPA1* (5 alleles; HLA‐DPA1*01:03; 36.1%) (Table S1). The association between two‐field HLA alleles and COVID‐19 severity was also investigated using logistic regression under a dominant model, adjusted for age and NAM ancestry. All HLA alleles with a minimum frequency of 0.01 were included in this analysis. Results suggested an increased risk of severe COVID‐19 for HLA‐DPB1*13:01 carriers (OR = 3.43, 95% CI = [1.08–12.05], *p* = 0.041), although the significance was lost after FDR correction for multiple comparisons (adjusted *p* > 0.05) (Table S3). The OR value was similar to that obtained for the HLA‐DPB1*13 group, suggesting that both analyses are reflecting the same effect.

Furthermore, for fine mapping, an omnibus test implemented in the MiDAS R package was applied to investigate amino acid positions strongly associated with COVID‐19 severity, using variations inferred from all available HLA allele calls. Table S4 shows the complete list of amino acid residues investigated. In this framework, the omnibus test serves as an initial screening step at the amino acid position level, followed by residue‐specific tests that aggregate all alleles carrying the same residue. Results of dominant models showed a borderline *p*‐value for the amino acid position 116 in the *HLA‐B* before FDR adjustment (*p* = 0.048; adjusted *p* = 0.98) (Table S4). Among the five possible amino acid residues observed at this position (serine, phenylalanine, leucine, tyrosine, and aspartic acid), the presence of serine was significantly associated with a protective effect against severe COVID‐19 (OR = 0.4774, 95% CI = [0.28–0.81], *p* = 0.0061) (Table S5), and the association remained significant after FDR adjustment (adjusted *p* = 0.0308). These results suggest that the alleles carrying serine at position 116 (HLA‐B*15:01, HLA‐B*15:03, HLA‐B*18:01, HLA‐B*35:01, HLA‐B*53:01, HLA‐B*58:01) are likely contributing to the protective effect associated with this residue (Table S6).

### 3.3 HLA Peptide‐Binding Affinity

To investigate the potential protective role of HLA‐B amino acid variants identified at position 116, we analysed the binding affinity of its associated allotypes to peptides derived from the SARS‐CoV‐2 Spike glycoprotein, responsible for viral entry mediation. This analysis was conducted exclusively on HLA‐B. Using NetMHCpan, we identified 73 peptides with strong binding affinity (IC50 < 50 nM) across the tested allotypes. Only HLA alleles with frequencies of at least 0.01 were considered for this analysis, as conducted for association analysis. A comprehensive table of peptide‐allotype interactions was generated to visualise binding patterns (Table S7). Notably, the peptide VASQSIIAY exhibited strong binding to 5 out of 7 allotypes (HLA‐B*15:01, HLA‐B*15:03, HLA‐B*35:01, HLA‐B*53:01, HLA‐B*58:01), suggesting a potential immunodominant character. Furthermore, the allotype HLA‐B*15:03 exhibited the highest number of peptides with strong binding affinity (*N* = 25/1265), indicating a broader capacity for antigen presentation. Together, these results suggest that HLA‐B*15:03 may possess an enhanced ability to recognise potentially immunodominant epitopes.

To explore the potential breadth of the protective effect associated with serine at position 116 of HLA‐B, the binding affinity analysis was also performed for peptides derived from the Spike protein of the Omicron BA.2.12.1 variant. As observed with the Wuhan strain, the peptide VASQSIIAY exhibited strong binding to 5 out of 7 allotypes (HLA‐B*15:01, HLA‐B*15:03, HLA‐B*35:01, HLA‐B*53:01, HLA‐B*58:01), reinforcing its potential immunodominant character even in the context of a variant of concern. Notably, HLA‐B*15:03 displayed the highest number of strong‐binding peptides (*N* = 27/1262), suggesting a preserved and possibly expanded antigen presentation capacity. These results are summarised in Table S8.

### 3.4 Association Between Single Nucleotide Variants at HLA Region and COVID‐19 Severity

In addition to the 6202 SNPs initially genotyped by microarray and retained after quality control, imputation was performed with high accuracy (*r* ^2^ ≥ 0.8), resulting in a final total of 14,519 variants. The association between each variant and COVID‐19 severity was assessed using logistic regression models under an additive model adjusted for age and NAM ancestry. Results of these analyses are shown in Figure S2. The variant rs78689026 (NC_000006.11:g.30740038G>A), located at the intronic region of the *HCG20* gene, showed the lowest *p*‐value and a trend towards an association with increased risk of severe COVID‐19 (OR = 3.05; 95% CI = 1.57–5.92; *p* = 0.001). However, these results were not statistically significant after adjustment for multiple comparisons (adjusted *p* = 0.43).

## 4 Discussion

In the context of COVID‐19, progression to severe disease is primarily driven by an exaggerated immune response [28]. Given the importance of the HLA region in triggering and modulating immune responses to viral infections, a fine‐mapping study was conducted to investigate the impact of the HLA region on severe COVID‐19 outcomes. Investigating the association of the HLA region with disease outcomes is, particularly, challenging due to its high polymorphism and strong LD in addition to the remarkable variation between populations, which may lead to population‐specific effects. As observed since the early stages of the pandemic [29], individuals with severe COVID‐19 in our cohort were predominantly older males, and presented with at least one comorbidity. The group with mild symptoms was matched to severe cases by sex and by the presence of at least one comorbidity (hypertension and/or diabetes) to minimise confounding. As expected for the Rio de Janeiro population, ancestry analysis revealed that proportions of European ancestry were the highest in both groups followed by African ancestry, while NAM ancestry represented the smallest proportion [30]. Following stepwise analysis, age and NAM ancestry were retained as covariates and incorporated into the logistic regression models to control for confounding.

Our findings suggest a risk effect for severe COVID‐19 in individuals carrying the HLA‐B*39 allele group, although this association did not remain significant after correction for multiple comparisons. Previous studies in Spanish and Ecuadorian populations have also reported an increased frequency of this allele group among COVID‐19 cases, supporting its possible relevance [14, 31]. The allele group HLA‐DPB1*13 and allele HLA‐DPB1*13:01 also showed a suggestive risk effect in our cohort, consistent with its reported involvement in susceptibility to other infections, such as Hepatitis B [32]. Contrary to our findings, a study in Italian individuals reported a protective effect of the HLA‐DPB1*13:01 allele against severe COVID‐19, suggesting population‐specific effects [15]. Given the small sample size, we were unable to perform haplotype analysis, which could have provided additional insights into the association of HLA alleles in our population.

Although initial infection typically occurs in the nasal epithelium, lung epithelial cells play a crucial role during the progression of respiratory infections such as COVID‐19, particularly in antigen presentation and immune activation [33]. Gene expression analyses, including validations in epithelial lung cell lines, have shown that *HLA‐B* is overexpressed in individuals infected with SARS‐CoV‐2 and is associated with disease susceptibility and severity. Although the mechanisms underlying *HLA‐B* overexpression remain unclear, a role for IFN‐β has been suggested [34]. In the present study, the presence of a serine at HLA‐B 116 amino acid position was associated with protection against severe COVID‐19 (OR = 0.47; 95% CI = 0.28–0.81; adjusted *p* = 0.031).

Residue 116 represents a critical site within the F pocket of HLA‐B, which anchors the C‐terminal end of the presented peptide [35]. In our in silico binding affinity analysis, the allotypes sharing Serine 116 (B*15:01, B*15:03, B*18:01, B*35:01, B*53:01, B*58:01) exhibited strong binding to SARS‐CoV‐2 Spike peptides, suggesting a potential role of this residue in enhancing viral antigen presentation efficiency. This potential effect appeared to be preserved for peptides derived from the Omicron BA.2.12.1 variant. Notably, HLA‐B*15:03 allotype displayed the highest number of strong‐binding peptides, reinforcing the notion that Serine 116 may contribute to the recognition of potentially immunodominant epitopes. Supporting this hypothesis, previous in silico analyses have shown that HLA‐B*15:03 strongly binds to peptides from SARS‐CoV‐2 and seasonal coronaviruses (OC43, HKU1, NL63 and 229E) [36], suggesting efficient immune activation and potential cross‐protective T‐cell immunity. Similarly, results of MHC multimer‐guided single‐cell RNA sequencing of SARS‐CoV‐2‐responsive T cells from vaccinated and infected donors of the United States showed that HLA‐B*15:01 presents the spike‐derived epitope NQKLIANQF, which cross‐reacts with common seasonal coronaviruses (HCoV‐OC43 and HCoV‐HKU1) [37]. In our analysis, the same allotypes showed stronger affinity to this peptide, as compared to allotypes B*15:03 and B*18:01.

The biological relevance of Serine 116 is also supported by functional evidence from other cohorts. In particular, studies in the United States associated HLA‐B*15:01 with asymptomatic COVID‐19 in individuals of European ancestry, in which pre‐existing memory T cells cross‐reacted with conserved epitopes from SARS‐CoV‐2 and seasonal coronaviruses (HCoV‐OC43 and HKU1) [38]. Functional analyses have also demonstrated that HLA‐B*15:01 is an immunodominant allele in CD8+ T cell responses to SARS‐CoV‐2, with higher recognition rates by CD8+ T cells in infected individuals compared to non‐infected controls [39]. Similarly, HLA‐B*35:01, which also harbours Serine 116 and showed strong binding to several SARS‐CoV‐2 epitopes, has been implicated in HIV infection, where amino acid changes at position 116 were associated with differences in peptide binding stability and infection outcomes [40, 41]. Although this allele did not reach significance in our dataset, its functional profile supports the role of Serine 116 variation in shaping antigen presentation and immune recognition, underscoring the relevance of epitopes preferentially presented by Serine 116‐carrying alleles in protective responses against SARS‐CoV‐2.

The epitope VASQSIIAY (687‐695), located in the furin cleavage site of SARS‐CoV‐2's Spike protein, has emerged as a promising immunogenic target for vaccine candidate design. A study focusing on Filipino and global variants identified VASQSIIAY as a conserved, highly antigenic epitope capable of eliciting robust CD8+ T‐cell responses [42]. Notably, its structural proximity to the superantigenic four‐residue insert (PRRA) in the S1/S2 boundary, critical for viral entry [43], underscores its functional relevance. In the present study, peptide VASQSIIAY showed strong binding to HLA alleles carrying Serine 116—both in the context of the Wuhan strain and the Omicron BA.2.12.1 variant—which was associated with protection against severe COVID‐19 respiratory outcomes. These data reinforce VASQSIIAY as a promising candidate for vaccine designs and highlight the importance of including genetically diverse populations to assess epitope immunological relevance across distinct genetic backgrounds.

Our study has some limitations. The relatively small sample size may have limited the statistical power to detect associations under a stringent FDR correction. Furthermore, the frequency‐matching strategy based on the presence of any major comorbidity, instead of specific conditions, constitutes an additional methodological constraint.

In summary, our study investigated the role of HLA genetic variability in COVID‐19 severity within an admixed Brazilian cohort, integrating allele and allele group level with peptide‐binding affinity analyses. SNP analyses were also performed, and no clear association signals were detected. We identified suggestive risk effects for HLA‐B*39 and HLA‐DPB1*13:01, along with a statistically significant protective association of serine at position 116 of HLA‐B, with enhanced binding affinity to immunodominant SARS‐CoV‐2 epitopes. Despite the sample size limitation, our comprehensive approach provides valuable insights into the role of the HLA complex in COVID‐19 severity within an admixed population. Further validation in larger cohorts is needed to clarify possible clinical and therapeutic implications.

## Author Contributions

Maria Aparecida da Silva Pereira, Terezinha Marta Pereira Pinto Castiñeiras, Amilcar Tanuri and Cynthia Chester Cardoso conceived the study. Mirella Carneiro dos Reis, Isabela de Carvalho Leitão, Rafael Mello Galliez, Shana Priscila Coutinho Barroso and Terezinha Marta Pereira Pinto Castiñeiras performed clinical diagnosis of the study participants and collected clinical and demographic data. Sâmila Natiane Ferreira, Cássia Cristina Alves Gonçalves, Isabela de Carvalho Leitão, Rafael Mello Galliez and Shana Priscila Coutinho Barroso collected the samples and performed molecular diagnosis of the study participants. Maria Aparecida da Silva Pereira, Sâmila Natiane Ferreira, Cássia Cristina Alves Gonçalves, Mirella Carneiro dos Reis, Carolina da Paz Zampier, Monique de Souza Almeida Lopes and Sheila Coelho Soares‐Lima conducted the experiments. Maria Aparecida da Silva Pereira, Artur Duque Rossi, Sheila Coelho Soares‐Lima, Carolina da Paz Zampier, Artur Duque Rossi and Cynthia Chester Cardoso analysed the data. Cynthia Chester Cardoso, Amilcar Tanuri and Terezinha Marta Pereira Pinto Castiñeiras financed the study. Maria Aparecida da Silva Pereira, Artur Duque Rossi, Orlando da Costa Ferreira Junior and Cynthia Chester Cardoso wrote the manuscript. All authors have read and agreed to the published version of the manuscript.

## Funding

This work was supported by Fundação Carlos Chagas Filho de Amparo à Pesquisa do Estado do Rio de Janeiro (FAPERJ), grant numbers E‐26/210.468/2021 (C.C.C.), E‐26/210.658/2021 (T.M.P.C.), E‐26/211.111/2021 and E‐26/210.178/2020 (A.T.) and E‐26/202.922/2023 (Postdoctoral fellowship to C.C.A.G.). This study was financed in part by the Coordenação de Aperfeiçoamento de Pessoal de Nível Superior—Brasil (CAPES)—Finance Code 001 (graduate scholarships to M.A.S.P. and S.N.F.).

## Ethics Statement

The study was approved by the Brazilian National Commission of Ethics in Research and by the institutional ethical review board from HNMD (protocol numbers 30161620.0.0000.5257 and 32382820.3.0000.5256, respectively).

## Consent

Written informed consent was obtained from all participants.

## Conflicts of Interest

The authors declare no conflicts of interest.

## Supporting information


**Box S1:** Stepwise forward logistic regression for covariates selection.
**Figure S1:** Overview of the study design and HLA association analyses.
**Figure S2:** *Manhattan plot* showing association analyses between single nucleotide variants at HLA region and COVID‐19 severity.
**Table S1:** Frequency of the 191 alleles imputed with high accuracy (*r* ^2^ ≥ 0.8).
**Table S2:** Association between HLA Classes I and II allelic groups and COVID‐19 severity.
**Table S3:** Association between carriers of each HLA allele from Classes I and II and COVID‐19 severity.
**Table S4:** Association between COVID‐19 severity and amino acid positions at HLA Classes I and II genes under dominant model.
**Table S5:** Association between each amino acid at HLA‐B 116 site and COVID‐19 severity and under dominant model.
**Table S6:** Alleles carrying each amino acid residue at HLA‐B 116 site.
**Table S7:** Strong‐binding peptides from the SARS‐CoV‐2 Spike protein (Wuhan strain) to HLA‐B allotypes carrying serine at position 116.
**Table S8:** Strong‐binding peptides from the SARS‐CoV‐2 Spike protein (Omicron BA.2.12.1 variant) to HLA‐B allotypes carrying serine at position 116.

## Data Availability

The data that support the findings of this study are available from the corresponding author upon reasonable request.

## References

[1] B. Hu , H. Guo , P. Zhou , and Z.‐L. Shi , “Characteristics of SARS‐CoV‐2 and COVID‐19,” Nature Reviews. Microbiology 19 (2021): 141–154. 33024307 10.1038/s41579-020-00459-7 PMC7537588

[2] Johns Hopkins University & Medicine , Mortality Analyses (Johns Hopkins Coronavirus Resource Center, 2023), https://coronavirus.jhu.edu/data/mortality.

[3] J. Zhang , X. Dong , G. Liu , and Y. Gao , “Risk and Protective Factors for COVID‐19 Morbidity, Severity, and Mortality,” Clinical Reviews in Allergy and Immunology 64 (2023): 90–107. 35044620 10.1007/s12016-022-08921-5 PMC8767775

[4] D. Ellinghaus , F. Degenhardt , L. Bujanda , et al., “Genomewide Association Study of Severe Covid‐19 With Respiratory Failure,” New England Journal of Medicine 383 (2020): NEJMoa2020283. 32558485 10.1056/NEJMoa2020283 PMC7315890

[5] A. C. Pereira , T. M. Bes , M. Velho , et al., “Genetic Risk Factors and COVID‐19 Severity in Brazil: Results From BRACOVID Study,” Human Molecular Genetics 31 (2022): 3021–3031. 35368071 10.1093/hmg/ddac045

[6] Mapping the Human Genetic Architecture of COVID‐19,” Nature 600 (2021): 472–477. 34237774 10.1038/s41586-021-03767-x PMC8674144

[7] B. Hu , S. Huang , and L. Yin , “The Cytokine Storm and COVID‐19,” Journal of Medical Virology 93 (2021): 250–256. 32592501 10.1002/jmv.26232 PMC7361342

[8] T. Shiina , K. Hosomichi , H. Inoko , and J. K. Kulski , “The HLA Genomic Loci Map: Expression, Interaction, Diversity and Disease,” Journal of Human Genetics 54 (2009): 15–39. 19158813 10.1038/jhg.2008.5

[9] R. Barquera , E. Collen , D. Di , et al., “Binding Affinities of 438 HLA Proteins to Complete Proteomes of Seven Pandemic Viruses and Distributions of Strongest and Weakest HLA Peptide Binders in Populations Worldwide,” HLA 96, no. 3 (2020): 277–298. 32475052 10.1111/tan.13956 PMC7300650

[10] V. Rao and N. Chandra , “In‐Silico Study of Influence of HLA Heterogeneity on CTL Responses Across Ethnicities to SARS‐CoV‐2,” Human Immunology 83 (2022): 797–802. 36229378 10.1016/j.humimm.2022.09.008 PMC9550298

[11] E. Pairo‐Castineira , S. Clohisey , L. Klaric , et al., “Genetic Mechanisms of Critical Illness in COVID‐19,” Nature 591 (2021): 92–98. 33307546 10.1038/s41586-020-03065-y

[12] E. Pairo‐Castineira , K. Rawlik , A. D. Bretherick , et al., “GWAS and Meta‐Analysis Identifies 49 Genetic Variants Underlying Critical COVID‐19,” Nature 617 (2023): 764–768. 37198478 10.1038/s41586-023-06034-3 PMC10208981

[13] P. Deb , K.‐E. Zannat , S. Talukder , A. H. Bhuiyan , J. MSA , and K. M. Saif‐Ur‐Rahman , “Association of HLA Gene Polymorphism With Susceptibility, Severity, and Mortality of COVID‐19: A Systematic Review,” HLA 99 (2022): 281–312. 35067002 10.1111/tan.14560

[14] A. Balas , M. Á. Moreno‐Hidalgo , F. de la Calle‐Prieto , et al., “Coronavirus‐19 Disease Risk and Protective Factors Associated With HLA/KIR Polymorphisms in Ecuadorian Patients Residing in Madrid,” Human Immunology 84 (2023): 571–577. 37777360 10.1016/j.humimm.2023.09.004

[15] T. D. J. Farias , S. Brugiapaglia , S. Croci , et al., “HLA‐DPB1*13:01 Associates With Enhanced, and KIR2DS4*001 With Diminished Protection From Developing Severe COVID‐19,” HLA 103 (2024): e15251. 37850268 10.1111/tan.15251 PMC10873037

[16] K. Nunes , M. Araújo Castro e Silva , M. R. Rodrigues , et al., “Admixture's Impact on Brazilian Population Evolution and Health,” Science 388 (2025): eadl3564. 40373151 10.1126/science.adl3564

[17] C. C. Chang , C. C. Chow , L. C. Tellier , S. Vattikuti , S. M. Purcell , and J. J. Lee , “Second‐Generation PLINK: Rising to the Challenge of Larger and Richer Datasets,” GigaScience 4 (2015): 7. 25722852 10.1186/s13742-015-0047-8 PMC4342193

[18] S. J. Schrodi , V. E. Garcia , C. Rowland , and H. B. Jones , “Pairwise Linkage Disequilibrium Under Disease Models,” European Journal of Human Genetics 15 (2007): 212–220. 17106449 10.1038/sj.ejhg.5201731

[19] A. T. Marees , H. de Kluiver , S. Stringer , et al., “A Tutorial on Conducting Genome‐Wide Association Studies: Quality Control and Statistical Analysis,” International Journal of Methods in Psychiatric Research 27 (2018): e1608. 29484742 10.1002/mpr.1608 PMC6001694

[20] D. H. Alexander , J. Novembre , and K. Lange , “Fast Model‐Based Estimation of Ancestry in Unrelated Individuals,” Genome Research 19 (2009): 1655–1664. 19648217 10.1101/gr.094052.109 PMC2752134

[21] Y. Luo , M. Kanai , W. Choi , et al., “A High‐Resolution HLA Reference Panel Capturing Global Population Diversity Enables Multi‐Ancestry Fine‐Mapping in HIV Host Response,” Nature Genetics 53 (2021): 1504–1516. 34611364 10.1038/s41588-021-00935-7 PMC8959399

[22] S. Das , L. Forer , S. Schönherr , et al., “Next‐Generation Genotype Imputation Service and Methods,” Nature Genetics 48 (2016): 1284–1287. 27571263 10.1038/ng.3656 PMC5157836

[23] Z. Bursac , C. H. Gauss , D. K. Williams , and D. W. Hosmer , “Purposeful Selection of Variables in Logistic Regression,” Source Code for Biology and Medicine 3 (2008): 17. 19087314 10.1186/1751-0473-3-17 PMC2633005

[24] M. Migdal , D. F. Ruan , W. F. Forrest , A. Horowitz , and C. Hammer , “MiDAS—Meaningful Immunogenetic Data at Scale,” PLoS Computational Biology 17 (2021): e1009131. 34228721 10.1371/journal.pcbi.1009131 PMC8284797

[25] B. Reynisson , B. Alvarez , S. Paul , B. Peters , and M. Nielsen , “NetMHCpan‐4.1 and NetMHCIIpan‐4.0: Improved Predictions of MHC Antigen Presentation by Concurrent Motif Deconvolution and Integration of MS MHC Eluted Ligand Data,” Nucleic Acids Research 48 (2020): W449–W454. 32406916 10.1093/nar/gkaa379 PMC7319546

[26] The UniProt Consortium , “UniProt: The Universal Protein Knowledgebase in 2025,” Nucleic Acids Research 53 (2025): D609–D617. 39552041 10.1093/nar/gkae1010 PMC11701636

[27] Y. Cao , A. Yisimayi , F. Jian , et al., “BA.2.12.1, BA.4 and BA.5 Escape Antibodies Elicited by Omicron Infection,” Nature 608 (2022): 593–602. 35714668 10.1038/s41586-022-04980-y PMC9385493

[28] M. Merad and J. C. Martin , “Pathological Inflammation in Patients With COVID‐19: A Key Role for Monocytes and Macrophages,” Nature Reviews. Immunology 20 (2020): 355–362. 10.1038/s41577-020-0331-4 PMC7201395 32376901

[29] M. A. Barek , M. A. Aziz , and M. S. Islam , “Impact of Age, Sex, Comorbidities and Clinical Symptoms on the Severity of COVID‐19 Cases: A Meta‐Analysis With 55 Studies and 10014 Cases,” Heliyon 6 (2020): e05684. 33344791 10.1016/j.heliyon.2020.e05684 PMC7737518

[30] S. D. J. Pena , F. R. Santos , and E. Tarazona‐Santos , “Genetic Admixture in Brazil,” American Journal of Medical Genetics. Part C, Seminars in Medical Genetics 184 (2020): 928–938. 33205899 10.1002/ajmg.c.31853

[31] L. Lorente , M. M. Martín , A. Franco , et al., “HLA Genetic Polymorphisms and Prognosis of Patients With COVID‐19,” Medicina Intensiva 45 (2021): 96–103. 10.1016/j.medin.2020.08.004 PMC7474921 38620408

[32] G. Ou , X. Liu , H. Xu , X. Ji , X. Liu , and J. Wang , “Variation and Expression of HLA‐DPB1 Gene in HBV Infection,” Immunogenetics 73 (2021): 253–261. 33710355 10.1007/s00251-021-01213-w

[33] W. Sungnak , N. Huang , C. Bécavin , et al., “SARS‐CoV‐2 Entry Factors Are Highly Expressed in Nasal Epithelial Cells Together With Innate Immune Genes,” Nature Medicine 26 (2020): 681–687. 10.1038/s41591-020-0868-6 PMC8637938 32327758

[34] Y. Zhang , Y. Sun , H. Zhu , et al., “Allelic Imbalance of HLA‐B Expression in Human Lung Cells Infected With Coronavirus and Other Respiratory Viruses,” European Journal of Human Genetics 30 (2022): 922–929. 35322240 10.1038/s41431-022-01070-5 PMC8940983

[35] V. Thammavongsa , G. Raghuraman , T. M. Filzen , K. L. Collins , and M. Raghavan , “HLA‐B44 Polymorphisms at Position 116 of the Heavy Chain Influence TAP Complex Binding via an Effect on Peptide Occupancy,” Journal of Immunology 177 (2006): 3150–3161. 10.4049/jimmunol.177.5.3150 16920953

[36] A. Nguyen , J. K. David , S. K. Maden , et al., “Human Leukocyte Antigen Susceptibility Map for Severe Acute Respiratory Syndrome Coronavirus 2,” Journal of Virology 94 (2020): e00510‐20. 32303592 10.1128/JVI.00510-20 PMC7307149

[37] A. A. Minervina , M. V. Pogorelyy , A. M. Kirk , et al., “SARS‐CoV‐2 Antigen Exposure History Shapes Phenotypes and Specificity of Memory CD8+ T Cells,” Nature Immunology 23 (2022): 781–790. 35383307 10.1038/s41590-022-01184-4 PMC9106845

[38] D. G. Augusto , L. D. Murdolo , D. S. M. Chatzileontiadou , et al., “A Common Allele of HLA Is Associated With Asymptomatic SARS‐CoV‐2 Infection,” Nature 620 (2023): 128–136. 37468623 10.1038/s41586-023-06331-x PMC10396966

[39] S. K. Saini , D. S. Hersby , T. Tamhane , et al., “SARS‐CoV‐2 Genome‐Wide T Cell Epitope Mapping Reveals Immunodominance and Substantial CD8+ T Cell Activation in COVID‐19 Patients,” Science Immunology 6 (2021): eabf7550. 33853928 10.1126/sciimmunol.abf7550 PMC8139428

[40] J. Huang , J. J. Goedert , E. J. Sundberg , et al., “HLA‐B*35‐Px–Mediated Acceleration of HIV‐1 Infection by Increased Inhibitory Immunoregulatory Impulses,” Journal of Experimental Medicine 206 (2009): 2959–2966. 20008523 10.1084/jem.20091386 PMC2806456

[41] C. A. Lobos , D. S. Chatzileontiadou , B. Sok , et al., “Molecular Insights Into the HLA‐B35 Molecules' Classification Associated With HIV Control,” Immunology and Cell Biology 102 (2024): 34–45. 37811811 10.1111/imcb.12698 PMC10952751

[42] M. C. Perono , J. Pasion , J. S. Nas , and F. I. Heralde , “Mapping T‐Cell Furin Cleavage Site Epitopes in SARS‐CoV‐2 Spike Protein Sequences From The Philippines and Global Variants,” Journal of Applied Pharmaceutical Science 14 (2024): 197–211.

[43] V. Deepthi , A. Sasikumar , K. P. Mohanakumar , and U. Rajamma , “Computationally Designed Multi‐Epitope Vaccine Construct Targeting the SARS‐CoV‐2 Spike Protein Elicits Robust Immune Responses In Silico,” Scientific Reports 15 (2025): 9562. 40108271 10.1038/s41598-025-92956-z PMC11923050

