# Supplementary material for: HLA‐B Serine 116 Confers Protection Against Severe COVID‐19 in a Cohort From Rio de Janeiro, Brazil
Source: HLA. 2025 Nov 28;106(6):e70479. doi: 10.1111/tan.70479 (PMC12662660; doi:10.1111/tan.70479)
Supplement: Supplementary file 1 — Box S1: Stepwise forward logistic regression for covariates selection. Figure S1: Overview of the study design and HLA association analyses. Figure S2: Manhattan plot showing association analyses between single nucleotide variants at HLA region and COVID‐19 severity. Table S1: Frequency of the 191 alleles imputed with high accuracy (r 2 ≥ 0.8). Table S2: Association between HLA Classes I and II allelic groups and COVID‐19 severity. Table S3: Association between carriers of each HLA allele from Classes I and II and COVID‐19 severity. Table S4: Association between COVID‐19 severity and amino acid positions at HLA Classes I and II genes under dominant model. Table S5: Association between each amino acid at HLA‐B 116 site and COVID‐19 severity and under dominant model. Table S6: Alleles carrying each amino acid residue at HLA‐B 116 site. Table S7: Strong‐binding peptides from the SARS‐CoV‐2 Spike protein (Wuhan strain) to HLA‐B allotypes carrying serine at position 116. Table S8: Strong‐binding peptides from the SARS‐CoV‐2 Spike protein (Omicron BA.2.12.1 variant) to HLA‐B allotypes carrying serine at position 116. [file TAN-106-e70479-s001.docx]

**Supporting information**

**Manuscript:** HLA-B Ser116 confers protection against severe COVID-19 in a cohort from Rio de Janeiro, Brazil

**Box S1: Stepwise forward logistic regression for covariates selection.**

| **Model** | **p-value** | **Model comparisons**  **(p-value)** | **Decision** |
| --- | --- | --- | --- |
| 1: Age only | 4.55 x 10^-8^ | Reference | Reference |
| 2: Age + NAM | 3.91 x 10^-11^ | Model 1 *vs* model 2 (p = 7.49 x 10^-6^) | Accepted |
| 3: Age + NAM + diabetes | 1.5 x 10-^10^ | Model 2 *vs* model 3  (p = 0.37) | Rejected: diabetes did not improve model fit |
| 4: Age + NAM + EUR | 3.91 x 10^-11^ | Model 2 x model 4  (p = 0.87) | Rejected: EUR did not improve model fit |

Variables were prioritized according to their p-values in the descriptive analysis (Table 1) and sequentially tested. Inclusion of Native American (NAM) ancestry significantly improved model fit, whereas diabetes and European ancestry (EUR) did not. P-values were derived from the Likelihood Ratio Test.

**Figure S1: Overview of the study design and HLA association analyses.**


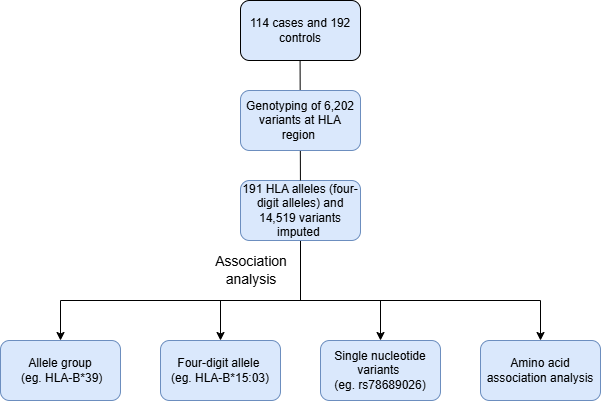


This schematic illustrates the study design aimed at identifying genetic associations with severe COVID-19. The case-control study design, including 114 severe COVID-19 cases and 192 individuals with mild symptoms. Genotyping was performed for 6,202 variants within the HLA region (chromosome 6: 25 - 35 Mb), followed by imputation of 191 four-digit HLA alleles and 14,519 additional variants. Based on this dataset, multiple independent association analyses were conducted at different resolution levels: allele group (e.g., HLA-B*39), four-digit alleles (e.g., HLA-B*15:03), single nucleotide variants (e.g., rs78689026), and amino acid residue positions.

**Table S1: Frequency of the 191 alleles imputed with high accuracy (r²≥0.8).**

| **Allele** | **N** | **Frequency** |
| --- | --- | --- |
| *HLA-A* |  |  |
| A*02:01  A*03:01  A*23:01  A*24:02  A*01:01  A*29:02  A*11:01  A*68:02  A*68:01  A*31:01  A*30:01  A*30:02  A*32:01  A*74:01  A*26:01  A*02:05  A*33:01  A*02:02  A*25:01  A*33:03  A*66:01  A*34:02  A*36:01  A*01:02  A*02:07  A*30:04  A*66:02  A*03:02  A*24:07  A*26:08  A*66:03  A*69:01  A*74:03  A*80:01 | 112  59  51  45  44  28  27  26  20  19  16  15  15  14  13  11  11  7  7  7  7  4  4  3  2  2  2  1  1  1  1  1  1  1 | 0.187  0.099  0.085  0.075  0.074  0.047  0.045  0.043  0.033  0.032  0.027  0.025  0.025  0.023  0.022  0.018  0.018  0.012  0.012  0.012  0.012  0.007  0.007  0.005  0.003  0.003  0.003  0.002  0.002  0.002  0.002  0.002  0.002  0.002 |
| *HLA-B* |  |  |
| B*07:02  B*35:01  B*44:03  B*51:01  B*14:02  B*53:01  B*18:01  B*08:01  B*44:02  B*50:01  B*49:01  B*15:01  B*15:03  B*58:01  B*13:02  B*42:01  B*38:01  B*40:01  B*52:01  B*57:01  B*58:02  B*35:03  B*40:02  B*15:10  B*27:05  B*37:01  B*41:01  B*45:01  B*39:06  B*57:03  B*15:17  B*35:02  B*35:08  B*81:01  B*27:03  B*55:01  B*14:01  B*40:04  B*07:05  B*15:16  B*27:02  B*48:02  B*73:01  B*35:12  B*39:10  B*41:02  B*47:01 | 40  39  36  36  30  29  25  24  22  20  17  13  12  12  11  11  10  10  10  10  10  9  9  7  7  7  7  7  6  6  5  5  5  5  4  4  3  3  2  2  2  2  2  1  1  1  1 | 0.067  0.065  0.060  0.060  0.050  0.048  0.042  0.040  0.037  0.033  0.028  0.022  0.020  0.020  0.018  0.018  0.017  0.017  0.017  0.017  0.017  0.015  0.015  0.012  0.012  0.012  0.012  0.012  0.010  0.010  0.008  0.008  0.008  0.008  0.007  0.007  0.005  0.005  0.003  0.003  0.003  0.003  0.003  0.002  0.002  0.002  0.002 |
| *HLA-C* |  |  |
| C*04:01  C*07:01  C*07:02  C*06:02  C*02:02  C*08:02  C*03:04  C*16:01  C*12:03  C*05:01  C*03:03  C*17:01  C*15:02  C*01:02  C*14:02  C*03:02  C*18:01  C*07:04  C*12:02  C*15:05  C*16:02  C*08:04  C*14:03  C*04:07  C*15:04  C*15:09  C*16:04 | 111  67  62  60  38  34  32  30  27  25  17  15  14  11  8  7  7  4  4  4  3  2  2  1  1  1  1 | 0.186  0.112  0.104  0.100  0.064  0.057  0.054  0.050  0.045  0.042  0.028  0.025  0.023  0.018  0.013  0.012  0.012  0.007  0.007  0.007  0.005  0.003  0.003  0.002  0.002  0.002  0.002 |
| *HLA-DRB1* |  |  |
| DRB1*07:01  DRB1*03:01  DRB1*13:01  DRB1*01:01  DRB1*11:01  DRB1*15:03  DRB1*15:01  DRB1*13:02  DRB1*01:02  DRB1*03:02  DRB1*14:01  DRB1*11:02  DRB1*04:05  DRB1*08:01  DRB1*04:02  DRB1*12:01  DRB1*08:04  DRB1*13:03  DRB1*16:02  DRB1*10:01  DRB1*01:03  DRB1*04:01  DRB1*11:03  DRB1*11:04  DRB1*16:01  DRB1*09:01  DRB1*13:05  DRB1*04:06  DRB1*08:03  DRB1*13:04  DRB1*14:04  DRB1*14:05  DRB1*15:02 | 68  54  39  37  32  32  30  28  23  16  14  13  12  11  10  10  8  8  8  7  6  6  6  6  6  5  5  3  2  2  1  1  1 | 0.114  0.090  0.065  0.062  0.054  0.054  0.050  0.047  0.038  0.027  0.023  0.022  0.020  0.018  0.017  0.017  0.013  0.013  0.013  0.012  0.010  0.010  0.010  0.010  0.010  0.008  0.008  0.005  0.003  0.003  0.002  0.002  0.002 |
| *HLA-DQA1* |  |  |
| DQA1*01:01  DQA1*01:02  DQA1*01:03  DQA1*02:01  DQA1*03:01  DQA1*04:01  DQA1*05:01  DQA1*06:01 | 147  116  96  77  69  49  39  3 | 0.246  0.194  0.161  0.129  0.115  0.082  0.065  0.005 |
| *HLA-DQB1* |  |  |
| DQB1*02:01  DQB1*03:01  DQB1*05:01  DQB1*06:02  DQB1*04:02  DQB1*03:02  DQB1*06:03  DQB1*06:04  DQB1*05:03  DQB1*03:03  DQB1*05:02  DQB1*06:09  DQB1*03:04  DQB1*06:01 | 132  105  85  67  50  45  39  22  15  14  13  9  1  1 | 0.221  0.176  0.142  0.112  0.084  0.075  0.065  0.037  0.025  0.023  0.022  0.015  0.002  0.002 |
| *HLA-DPA1* |  |  |
| DPA1*01:03  DPA1*02:01  DPA1*02:02  DPA1*03:03  DPA1*04:01 | 216  103  45  19  3 | 0.361  0.172  0.075  0.032  0.005 |
| HLA-DPB1 |  |  |
| DPB1*04:01  DPB1*02:01  DPB1*04:02  DPB1*01:01  DPB1*03:01  DPB1*17:01  DPB1*10:01  DPB1*13:01  DPB1*11:01  DPB1*14:01  DPB1*05:01  DPB1*18:01  DPB1*06:01  DPB1*02:02  DPB1*15:01  DPB1*16:01  DPB1*23:01  DPB1*29:01  DPB1*39:01  DPB1*19:01  DPB1*20:01  DPB1*30:01  DPB1*40:01 | 150  88  87  61  47  23  16  14  13  13  9  8  6  5  4  3  3  3  3  2  2  1  1 | 0.251  0.147  0.145  0.102  0.079  0.038  0.027  0.023  0.022  0.022  0.015  0.013  0.010  0.008  0.007  0.005  0.005  0.005  0.005  0.003  0.003  0.002  0.002 |

Abbreviations: N, *N*: Individuals carrying the allele.

**Table S2: Association between HLA class I and II allelic groups and COVID-19 severity.**

|  | **Mild**  **symptoms** | | **Severe**  **disease** | |  |  |  | **_95%_CI** | |
| --- | --- | --- | --- | --- | --- | --- | --- | --- | --- |
| **HLA group** | **N** | % | **N** | % | ***p-value*** | **FDR** | **Odds ratio** | **Inf** | **Sup** |
| *HLA-A* |  |  |  |  |  |  |  |  |  |
| \| A*36 \| \| --- \| \| A*02 \| \| A*33 \| \| A*03 \| \| A*11 \| \| A*01 \| \| A*68 \| \| A*30 \| \| A*25 \| \| A*24 \| \| A*34 \| \| A*66 \| \| A*29 \| \| A*26 \| \| A*31 \| \| A*32 \| \| A*23 \| \| A*74 \| \| Rare \| \| \| Missing \| \| | \| 1 \| \| --- \| \| 82 \| \| 9 \| \| 35 \| \| 19 \| \| 30 \| \| 26 \| \| 23 \| \| 5 \| \| 26 \| \| 3 \| \| 7 \| \| 16 \| \| 10 \| \| 10 \| \| 9 \| \| 32 \| \| 9 \| \| 1 \| \| \| 31 \| \| | \| 0.26 \| \| --- \| \| 21.35 \| \| 2.34 \| \| 9.11 \| \| 4.95 \| \| 7.81 \| \| 6.77 \| \| 5.99 \| \| 1.30 \| \| 6.77 \| \| 0.78 \| \| 1.82 \| \| 4.17 \| \| 2.60 \| \| 2.60 \| \| 2.34 \| \| 8.33 \| \| 2.34 \| \| 0.26 \| \| 8.07 \| | \| 3 \| \| --- \| \| 39 \| \| 9 \| \| 25 \| \| 7 \| \| 15 \| \| 20 \| \| 11 \| \| 2 \| \| 20 \| \| 2 \| \| 3 \| \| 10 \| \| 5 \| \| 10 \| \| 6 \| \| 19 \| \| 6 \| \| 1 \| \| 15 \| | \| 1.32 \| \| --- \| \| 17.1 \| \| 3.95 \| \| 10.96 \| \| 3.07 \| \| 6.58 \| \| 8.77 \| \| 4.82 \| \| 0.88 \| \| 8.77 \| \| 0.88 \| \| 1.32 \| \| 4.39 \| \| 2.19 \| \| 4.39 \| \| 2.63 \| \| 8.33 \| \| 2.63 \| \| 0.44 \| \| 6.58 \| | \| 0.049 \| \| --- \| \| 0.153 \| \| 0.163 \| \| 0.197 \| \| 0.227 \| \| 0.332 \| \| 0.391 \| \| 0.393 \| \| 0.574 \| \| 0.589 \| \| 0.607 \| \| 0.673 \| \| 0.718 \| \| 0.876 \| \| 0.922 \| \| 0.927 \| \| 0.942 \| \| 0.974 \| \| NA \| \| NA \| | \| 0.908 \| \| --- \| \| 0.908 \| \| 0.908 \| \| 0.908 \| \| 0.908 \| \| 0.983 \| \| 0.983 \| \| 0.983 \| \| 0.987 \| \| 0.987 \| \| 0.987 \| \| 0.987 \| \| 0.987 \| \| 0.987 \| \| 0.987 \| \| 0.987 \| \| 0.987 \| \| 0.987 \| \| NA \| \| NA \| | \| 12.35 \| \| --- \| \| 0.67 \| \| 2.07 \| \| 1.51 \| \| 0.55 \| \| 0.69 \| \| 1.35 \| \| 0.69 \| \| 0.58 \| \| 1.20 \| \| 1.72 \| \| 0.73 \| \| 0.84 \| \| 0.91 \| \| 1.05 \| \| 0.94 \| \| 1.02 \| \| 1.01 \| \| NA \| \| NA \| | \| 1.00 \| \| --- \| \| 0.4 \| \| 0.74 \| \| 0.80 \| \| 0.21 \| \| 0.32 \| \| 0.67 \| \| 0.30 \| \| 0.08 \| \| 0.60 \| \| 0.21 \| \| 0.17 \| \| 0.33 \| \| 0.28 \| \| 0.37 \| \| 0.29 \| \| 0.52 \| \| 0.32 \| \| NA \| \| NA \| | \| 151.42 \| \| --- \| \| 1.15 \| \| 5.76 \| \| 2.82 \| \| 1.44 \| \| 1.45 \| \| 2.69 \| \| 1.58 \| \| 3.85 \| \| 2.41 \| \| 13.60 \| \| 3.04 \| \| 2.14 \| \| 2.93 \| \| 2.94 \| \| 3.08 \| \| 2 \| \| 3.15 \| \| NA \| \| NA \| |
| *HLA-B* |  |  |  |  |  |  |  |  |  |
| \| B*39 \| \| --- \| \| B*08 \| \| B*49 \| \| B*53 \| \| B*51 \| \| B*57 \| \| B*40 \| \| B*55 \| \| B*41 \| \| B*38 \| \| B*27 \| \| B*07 \| \| B*14 \| \| B*52 \| \| B*18 \| \| B*45 \| \| B*50 \| \| B*13 \| \| B*37 \| \| B*44 \| \| B*42 \| \| B*35 \| \| B*15 \| \| B*81 \| \| B*58 \| \| Rare \| \| \| Missin \| \| | \| 7 \| \| --- \| \| 20 \| \| 12 \| \| 20 \| \| 22 \| \| 9 \| \| 10 \| \| 3 \| \| 4 \| \| 8 \| \| 9 \| \| 25 \| \| 20 \| \| 8 \| \| 18 \| \| 4 \| \| 15 \| \| 9 \| \| 5 \| \| 34 \| \| 9 \| \| 37 \| \| 25 \| \| 3 \| \| 15 \| \| 2 \| \| 31 \| | \| 1.82 \| \| --- \| \| 5.21 \| \| 3.13 \| \| 5.21 \| \| 5.73 \| \| 2.34 \| \| 2.60 \| \| 0.78 \| \| 1.04 \| \| 2.08 \| \| 2.34 \| \| 6.51 \| \| 5.21 \| \| 2.08 \| \| 4.69 \| \| 1.04 \| \| 3.91 \| \| 2.34 \| \| 1.30 \| \| 8.85 \| \| 2.34 \| \| 9.64 \| \| 6.51 \| \| 0.78 \| \| 3.91 \| \| 0.52 \| \| 8.07 \| | \| 13 \| \| --- \| \| 5 \| \| 5 \| \| 8 \| \| 16 \| \| 5 \| \| 14 \| \| 1 \| \| 4 \| \| 2 \| \| 5 \| \| 20 \| \| 14 \| \| 3 \| \| 7 \| \| 3 \| \| 8 \| \| 3 \| \| 2 \| \| 21 \| \| 4 \| \| 23 \| \| 17 \| \| 2 \| \| 9 \| \| 3 \|  \| \| 11 \|  \| | \| 5.70 \| \| --- \| \| 2.19 \| \| 2.19 \| \| 3.51 \| \| 7.02 \| \| 2.19 \| \| 6.14 \| \| 0.44 \| \| 1.75 \| \| 0.88 \| \| 2.19 \| \| 8.77 \| \| 6.14 \| \| 1.32 \| \| 3.07 \| \| 1.32 \| \| 3.51 \| \| 1.32 \| \| 0.88 \| \| 9.21 \| \| 1.75 \| \| 10.09 \| \| 7.46 \| \| 0.88 \| \| 3.95 \| \| 1.32 \| \| 4.82 \| | \| 0.024 \| \| --- \| \| 0.173 \| \| 0.207 \| \| 0.217 \| \| 0.238 \| \| 0.277 \| \| 0.342 \| \| 0.365 \| \| 0.369 \| \| 0.382 \| \| 0.412 \| \| 0.462 \| \| 0.468 \| \| 0.515 \| \| 0.516 \| \| 0.644 \| \| 0.658 \| \| 0.749 \| \| 0.782 \| \| 0.8 \| \| 0.804 \| \| 0.892 \| \| 0.917 \| \| 0.943 \| \| 0.946 \| \| NA \| \| NA \| | \| 0.672 \| \| --- \| \| 0.963 \| \| 0.963 \| \| 0.963 \| \| 0.963 \| \| 0.963 \| \| 0.963 \| \| 0.963 \| \| 0.963 \| \| 0.963 \| \| 0.963 \| \| 0.963 \| \| 0.963 \| \| 0.963 \| \| 0.963 \| \| 0.988 \| \| 0.988 \| \| 0.988 \| \| 0.988 \| \| 0.988 \| \| 0.988 \| \| 0.988 \| \| 0.988 \| \| 0.988 \| \| 0.988 \| \| NA \| \| NA \| | \| 3.26 \| \| --- \| \| 0.48 \| \| 0.47 \| \| 0.57 \| \| 1.56 \| \| 0.47 \| \| 1.59 \| \| 0.3 \| \| 2.05 \| \| 0.49 \| \| 0.61 \| \| 1.3 \| \| 1.33 \| \| 0.59 \| \| 0.72 \| \| 1.48 \| \| 1.24 \| \| 0.8 \| \| 0.78 \| \| 1.09 \| \| 0.85 \| \| 1.05 \| \| 1.04 \| \| 1.07 \| \| 0.97 \| \| NA \|  \| \| NA \|  \| | \| 1.17 \| \| --- \| \| 0.16 \| \| 0.14 \| \| 0.23 \| \| 0.74 \| \| 0.12 \| \| 0.61 \| \| 0.02 \| \| 0.43 \| \| 0.1 \| \| 0.18 \| \| 0.64 \| \| 0.61 \| \| 0.12 \| \| 0.27 \| \| 0.28 \| \| 0.47 \| \| 0.2 \| \| 0.14 \| \| 0.57 \| \| 0.24 \| \| 0.55 \| \| 0.5 \| \| 0.17 \| \| 0.39 \| \| NA \| \| NA \| | \| 9.13 \| \| --- \| \| 1.39 \| \| 1.53 \| \| 1.4 \| \| 3.26 \| \| 1.83 \| \| 4.15 \| \| 4.09 \| \| 9.8 \| \| 2.42 \| \| 2.01 \| \| 2.64 \| \| 2.92 \| \| 2.9 \| \| 1.94 \| \| 7.92 \| \| 3.26 \| \| 3.23 \| \| 4.45 \| \| 2.08 \| \| 3.07 \| \| 1.97 \| \| 2.14 \| \| 6.78 \| \| 2.41 \| \| NA \| \| NA \| |
| *HLA-C* |  |  |  |  |  |  |  |  |  |
| \| C*18 \| \| --- \| \| C*06 \| \| C*15 \| \| C*12 \| \| C*02 \| \| C*14 \| \| C*01 \| \| C*03 \| \| C*04 \| \| C*07 \| \| C*08 \| \| C*17 \| \| C*05 \| \| C*16 \| \| Missing \| | \| 2 \| \| --- \| \| 40 \| \| 10 \| \| 19 \| \| 27 \| \| 5 \| \| 4 \| \| 30 \| \| 65 \| \| 73 \| \| 22 \| \| 9 \| \| 16 \| \| 21 \| \| 41 \| | \| 0.52 \| \| --- \| \| 10.42 \| \| 2.60 \| \| 4.95 \| \| 7.03 \| \| 1.30 \| \| 1.04 \| \| 7.81 \| \| 16.93 \| \| 19.01 \| \| 5.73 \| \| 2.34 \| \| 4.17 \| \| 5.47 \| \| 10.68 \| | \| 5 \| \| --- \| \| 17 \| \| 9 \| \| 12 \| \| 12 \| \| 5 \| \| 7 \| \| 25 \| \| 37 \| \| 47 \| \| 13 \| \| 6 \| \| 9 \| \| 12 \| \| 12 \| | \| 2.19 \| \| --- \| \| 7.46 \| \| 3.95 \| \| 5.26 \| \| 5.26 \| \| 2.19 \| \| 3.07 \| \| 10.96 \| \| 16.23 \| \| 20.61 \| \| 5.70 \| \| 2.63 \| \| 3.95 \| \| 5.26 \| \| 5.26 \| | \| 0.084 \| \| --- \| \| 0.255 \| \| 0.281 \| \| 0.428 \| \| 0.501 \| \| 0.617 \| \| 0.688 \| \| 0.694 \| \| 0.707 \| \| 0.782 \| \| 0.788 \| \| 0.827 \| \| 0.885 \| \| 0.904 \| \| NA \| | \| 0.904 \| \| --- \| \| 0.904 \| \| 0.904 \| \| 0.904 \| \| 0.904 \| \| 0.904 \| \| 0.904 \| \| 0.904 \| \| 0.904 \| \| 0.904 \| \| 0.904 \| \| 0.904 \| \| 0.904 \| \| 0.904 \|   NA | \| 4.41 \| \| --- \| \| 0.67 \| \| 1.75 \| \| 1.39 \| \| 0.77 \| \| 1.42 \| \| 1.35 \| \| 1.14 \| \| 0.9 \| \| 1.08 \| \| 1.11 \| \| 1.14 \| \| 0.93 \| \| 1.05 \| \| NA \| | \| 0.82 \| \| --- \| \| 0.34 \| \| 0.63 \| \| 0.62 \| \| 0.35 \| \| 0.36 \| \| 0.31 \| \| 0.59 \| \| 0.53 \| \| 0.64 \| \| 0.51 \| \| 0.36 \| \| 0.36 \| \| 0.47 \| \| NA \| | \| 23.88 \| \| --- \| \| 1.33 \| \| 4.86 \| \| 3.12 \| \| 1.66 \| \| 5.63 \| \| 5.91 \| \| 2.2 \| \| 1.54 \| \| 1.81 \| \| 2.43 \| \| 3.6 \| \| 2.4 \| \| 2.36 \| \| NA \| |
| *HLA-DRB1* |  |  |  |  |  |  |  |  |  |
| \| DRB1*12 \| \| --- \| \| DRB1*08 \| \| DRB1*07 \| \| DRB1*04 \| \| DRB1*10 \| \| DRB1*01 \| \| DRB1*15 \| \| DRB1*11 \| \| DRB1*09 \| \| DRB1*13 \| \| DRB1*14 \| \| DRB1*16 \| \| DRB1*03 \| \| Missing \| | \| 10 \| \| --- \| \| 19 \| \| 49 \| \| 32 \| \| 3 \| \| 38 \| \| 40 \| \| 38 \| \| 4 \| \| 57 \| \| 11 \| \| 7 \| \| 44 \| \| 32 \| | \| 2.60 \| \| --- \| \| 4.95 \| \| 12.76 \| \| 8.33 \| \| 0.78 \| \| 9.90 \| \| 10.42 \| \| 9.90 \| \| 1.04 \| \| 14.84 \| \| 2.86 \| \| 1.82 \| \| 11.46 \| \| 8.33 \| | \| 2 \| \| --- \| \| 19 \| \| 24 \| \| 26 \| \| 5 \| \| 28 \| \| 18 \| \| 20 \| \| 3 \| \| 31 \| \| 9 \| \| 6 \| \| 21 \| \| 16 \| \| \|  \| | \| 0.88 \| \| --- \| \| 8.33 \| \| 10.53 \| \| 11.40 \| \| 2.19 \| \| 12.28 \| \| 7.89 \| \| 8.77 \| \| 1.32 \| \| 13.60 \| \| 3.95 \| \| 2.63 \| \| 9.21 \| \| 7.02 \| | \| 0.132 \| \| --- \| \| 0.236 \| \| 0.277 \| \| 0.296 \| \| 0.395 \| \| 0.403 \| \| 0.455 \| \| 0.483 \| \| 0.6 \| \| 0.639 \| \| 0.8 \| \| 0.926 \| \| 0.95 \| \| NA \| | \| 0.785 \| \| --- \| \| 0.785 \| \| 0.785 \| \| 0.785 \| \| 0.785 \| \| 0.785 \| \| 0.785 \| \| 0.785 \| \| 0.831 \| \| 0.831 \| \| 0.945 \| \| 0.95 \| \| 0.95 \| \| NA \| | \| 0.27 \| \| --- \| \| 1.56 \| \| 0.71 \| \| 1.4 \| \| 1.99 \| \| 1.3 \| \| 0.78 \| \| 0.79 \| \| 0.64 \| \| 0.87 \| \| 1.14 \| \| 0.94 \| \| 0.98 \| \| NA \| | \| 0.05 \| \| --- \| \| 0.75 \| \| 0.39 \| \| 0.74 \| \| 0.41 \| \| 0.7 \| \| 0.4 \| \| 0.42 \| \| 0.12 \| \| 0.49 \| \| 0.41 \| \| 0.26 \| \| 0.53 \| \| NA \| | \| 1.49 \| \| --- \| \| 3.27 \| \| 1.31 \| \| 2.65 \| \| 9.75 \| \| 2.39 \| \| 1.5 \| \| 1.52 \| \| 3.34 \| \| 1.53 \| \| 3.14 \| \| 3.46 \| \| 1.83 \| \| NA \| |
| *HLA-DQA1* |  |  |  |  |  |  |  |  |  |
| \| DQA1*06 \| \| --- \| \| DQA1*05 \| \| DQA1*02 \| \| DQA1*03 \| \| DQA1*04 \| \| DQA1*01 \| \| Missing \| | \| 1 \| \| --- \| \| 82 \| \| 49 \| \| 39 \| \| 28 \| \| 127 \| \| 58 \| | \| 0.26 \| \| --- \| \| 21.35 \| \| 12.76 \| \| 10.16 \| \| 7.29 \| \| 33.07 \| \| 15.10 \| | \| 2 \| \| --- \| \| 44 \| \| 25 \| \| 30 \| \| 19 \| \| 76 \| \| 32 \| | \| 0.88 \| \| --- \| \| 19.30 \| \| 10.96 \| \| 13.16 \| \| 8.33 \| \| 33.33 \| \| 14.04 \| | \| 0.256 \| \| --- \| \| 0.277 \| \| 0.347 \| \| 0.533 \| \| 0.601 \| \| 1 \| \| NA \| | \| 0.694 \| \| --- \| \| 0.694 \| \| 0.694 \| \| 0.721 \| \| 0.721 \| \| 1 \| \| NA \| | \| 4.11 \| \| --- \| \| 0.75 \| \| 0.75 \| \| 1.21 \| \| 1.2 \| \| 1 \| \| NA \| | \| 0.36 \| \| --- \| \| 0.45 \| \| 0.41 \| \| 0.67 \| \| 0.6 \| \| 0.58 \| \| NA \| | \| 47.17 \| \| --- \| \| 1.26 \| \| 1.37 \| \| 2.19 \| \| 2.41 \| \| 1.73 \| \| NA \| |
| *HLA-DQB1* |  |  |  |  |  |  |  |  |  |
| \| DQB1*04 \| \| --- \| \| DQB1*03 \| \| DQB1*02 \| \| DQB1*05 \| \| DQB1*06 \| \| Missing \| | \| 26 \| \| --- \| \| 88 \| \| 81 \| \| 61 \| \| 81 \| \| 47 \| | \| 6.77 \| \| --- \| \| 22.92 \| \| 21.09 \| \| 15.89 \| \| 21.09 \| \| 12.24 \| | \| 22 \| \| --- \| \| 54 \| \| 40 \| \| 42 \| \| 41 \| \| 29 \| | \| 9.65 \| \| --- \| \| 23.68 \| \| 17.54 \| \| 18.42 \| \| 17.98 \| \| 12.72 \| | \| 0.178 \| \| --- \| \| 0.38 \| \| 0.394 \| \| 0.662 \| \| 0.665 \| \| NA \| | \| 0.657 \| \| --- \| \| 0.657 \| \| 0.657 \| \| 0.665 \| \| 0.665 \| \| NA \| | \| 1.6 \| \| --- \| \| 0.79 \| \| 0.79 \| \| 1.13 \| \| 0.89 \| \| NA \| | \| 0.81 \| \| --- \| \| 0.48 \| \| 0.47 \| \| 0.66 \| \| 0.53 \| \| NA \| | \| 3.18 \| \| --- \| \| 1.33 \| \| 1.35 \| \| 1.92 \| \| 1.5 \| \| NA \| |
| *HLA-DPA1* |  |  |  |  |  |  |  |  |  |
| \| DPA1*03 \| \| --- \| \| DPA1*01 \| \| DPA1*02 \| \| DPA1*04 \| \| Missing \| | \| 12 \| \| --- \| \| 105 \| \| 82 \| \| 2 \| \| 183 \| | \| 3.13 \| \| --- \| \| 27.34 \| \| 21.35 \| \| 0.52 \| \| 47.66 \| | \| 10 \| \| --- \| \| 61 \| \| 51 \| \| 1 \| \| 105 \| | \| 4.39 \| \| --- \| \| 26.75 \| \| 22.37 \| \| 0.44 \| \| 46.05 \| | \| 0.508 \| \| --- \| \| 0.607 \| \| 0.933 \| \| 0.976 \| \| NA \| | \| 0.976 \| \| --- \| \| 0.976 \| \| 0.976 \| \| 0.976 \| \| NA \| | \| 1.38 \| \| --- \| \| 0.87 \| \| 1.02 \| \| 0.96 \| \| NA \| | \| 0.53 \| \| --- \| \| 0.52 \| \| 0.61 \| \| 0.08 \| \| NA \| | \| 3.57 \| \| --- \| \| 1.46 \| \| 1.7 \| \| 11.15 \| \| NA \| |
| HLA-DPB1 |  |  |  |  |  |  |  |  |  |
| DPB1*13  DPB1*18  DPB1*01  DPB1*14  DPB1*17  DPB1*10  DPB1*15  DPB1*02  DPB1*05  DPB1*19  DPB1*03  DPB1*29  DPB1*06  DPB1*23  DPB1*04  DPB1*39  DPB1*11  Rare  Missing | 5  6  40  9  16  8  2  55  7  1  26  1  3  1  112  2  8  6  76 | 1.3  1.56  10.42  2.34  4.17  2.08  0.52  14.32  1.82  0.26  6.77  0.26  0.78  0.26  29.17  0.52  2.08  1.56  19.79 | 9  1  20  4  7  8  1  24  2  1  18  2  3  2  76  1  5  1  43 | 3.95  0.44  8.77  1.75  3.07  3.51  0.44  10.53  0.88  0.44  7.89  0.88  1.32  0.88  33.33  0.44  2.19  0.44  18.86 | 0.041  0.162  0.233  0.25  0.326  0.339  0.484  0.512  0.567  0.604  0.624  0.653  0.674  0.678  0.787  0.811  0.885  NA  NA | 0.861  0.988  0.988  0.988  0.988  0.988  0.988  0.988  0.988  0.988  0.988  0.988  0.988  0.988  0.988  0.988  0.988  NA  NA | 3.43  0.22  0.67  0.44  0.61  1.71  0.41  0.82  0.61  2.09  1.19  1.76  1.44  1.76  1.08  1.36  0.91  NA  NA | 1.05  0.03  0.35  0.11  0.23  0.57  0.03  0.45  0.12  0.13  0.59  0.15  0.26  0.12  0.63  0.11  0.27  NA  NA | 11.16  1.85  1.29  1.8  1.63  5.1  4.98  1.49  3.27  34.01  2.44  20.64  7.81  25.73  1.83  16.52  3.09  NA  NA |

Allele group frequencies and association analysis with COVID-19 severity under a dominant model.

Sample size: 192 severe cases (384 alleles) and 114 subjects with mild symptoms (228 alleles). Rows labeled as Missing indicate individuals without valid genotype calls, and rows labeled as Rare correspond to the number of alleles excluded from association analysis due to low frequency (< 1% in total cohort). P-values obtained from logistic regression adjusted for age (continuous) and Native American ancestry.

Abbreviations: N, number of alleles; %, frequency; OR, Odds ratio; CI, Confidence Interval; Inf, Inferior; Sup, Superior; NA, not available..

**Table S3: Association between carriers of each HLA allele from classes I and II and COVID-19 severity.**

|  | **Mild symptoms** | | **Severe**  **disease** | |  |  |  | **_95%_CI** | |
| --- | --- | --- | --- | --- | --- | --- | --- | --- | --- |
| **HLA allele** | **N** | % | **N** | % | ***p-value*** | **FDR** | **Odds ratio** | **Inf** | **Sup** |
| *HLA-A* |  |  |  |  |  |  |  |  |  |
| A*02:01  A*03:01  A*11:01  A*33:03  A*01:01  A*33:01  A*02:02  A*30:01  A*24:02  A*25:01  A*74:01  A*68:01  A*30:02  A*29:02  A*02:05  A*31:01  A*23:01  A*26:01  A*68:02  A*32:01  A*66:01  Missing | 70  34  19  3  28  6  5  12  23  5  9  10  9  16  7  10  31  8  16  9  4  50 | 18.23  8.85  4.95  0.78  7.29  1.56  1.30  3.13  5.99  1.30  2.34  2.60  2.34  4.17  1.82  2.60  8.07  2.08  4.17  2.34  1.04  13.02 | 30  24  7  4  13  5  2  4  18  2  5  8  6  10  4  9  19  5  10  6  3  34 | 13.16  10.53  3.07  1.75  5.70  2.19  0.88  1.75  7.89  0.88  2.19  3.51  2.63  4.39  1.75  3.95  8.33  2.19  4.39  2.63  1.32  14.91 | 0.094  0.17  0.228  0.247  0.328  0.401  0.446  0.461  0.558  0.574  0.67  0.675  0.703  0.719  0.767  0.804  0.856  0.862  0.889  0.928  0.946  NA | 0.967  0.967  0.967  0.967  0.967  0.967  0.967  0.967  0.967  0.967  0.967  0.967  0.967  0.967  0.967  0.967  0.967  0.967  0.967  0.967  0.969  NA | 0.62  1.56  0.56  2.76  0.68  1.71  0.49  0.63  1.24  0.58  0.77  1.25  0.80  0.84  1.22  0.87  1.06  1.11  1.07  0.95  1.06  NA | 0.35  0.82  0.20  0.50  0.30  0.47  0.06  0.16  0.60  0.07  0.22  0.43  0.24  0.32  0.29  0.29  0.54  0.31  0.42  0.27  0.20  NA | 1.08  2.95  1.39  17.14  1.45  6.05  2.85  2.00  2.55  3.37  2.46  3.50  2.45  2.10  4.49  2.52  2.07  3.67  2.59  3.02  4.98  NA |
| *HLA-B* |  |  |  |  |  |  |  |  |  |
| B*35:03  B*40:01  B*57:01  B*39:06  B*51:01  B*41:01  B*49:01  B*58:02  B*53:01  B*08:01  B*52:01  B*38:01  B*15:01  B*18:01  B*58:01  B*15:10  B*40:02  B*45:01  B*42:01  B*35:01  B*15:03  B*44:02  B*50:01  B*37:01  B*07:02  B*27:05  B*14:02  B*13:02  B*44:03  Missing | 1  3  7  2  19  3  12  8  20  19  8  8  9  18  7  4  5  4  8  25  7  13  14  5  23  4  19  8  23  78 | 0.26  0.78  1.82  0.52  4.95  0.78  3.13  2.08  5.21  4.95  2.08  2.08  2.34  4.69  1.82  1.04  1.30  1.04  2.08  6.51  1.82  3.39  3.65  1.30  5.99  1.04  4.95  2.08  5.99  20.31 | 7  7  2  4  14  4  5  2  8  5  1  2  4  7  5  3  4  3  3  12  5  9  6  2  16  3  11  3  12  59 | 3.07  3.07  0.88  1.75  6.14  1.75  2.19  0.88  3.51  2.19  0.44  0.88  1.75  3.07  2.19  1.32  1.75  1.32  1.32  5.26  2.19  3.95  2.63  0.88  7.02  1.32  4.82  1.32  5.26  25.88 | 0.007  0.116  0.12  0.14  0.196  0.206  0.208  0.214  0.218  0.238  0.286  0.382  0.387  0.517  0.571  0.594  0.617  0.644  0.645  0.651  0.67  0.719  0.765  0.783  0.794  0.895  0.896  0.898  0.956  NA | 0.846  0.967  0.967  0.967  0.967  0.967  0.967  0.967  0.967  0.967  0.967  0.967  0.967  0.967  0.967  0.967  0.967  0.967  0.967  0.967  0.967  0.967  0.967  0.967  0.967  0.967  0.967  0.967  0.972  NA | 21.54  3.40  0.23  3.74  1.68  3.01  0.47  0.36  0.57  0.52  0.32  0.49  0.55  0.72  1.43  1.54  0.69  1.48  0.71  0.83  1.31  1.19  1.17  0.78  1.11  0.89  1.06  0.91  1.02  NA | 3.31  0.78  0.03  0.69  0.76  0.54  0.13  0.05  0.22  0.16  0.02  0.07  0.13  0.25  0.39  0.28  0.15  0.25  0.14  0.37  0.36  0.44  0.39  0.11  0.52  0.15  0.45  0.18  0.45  NA | 433.25  18.02  1.23  28.01  3.70  18.19  1.45  1.55  1.35  1.44  1.82  2.07  1.98  1.87  4.95  7.50  2.98  8.04  2.78  1.80  4.45  3.09  3.20  4.05  2.32  4.82  2.41  3.55  2.23  NA |
| *HLA-C* |  |  |  |  |  |  |  |  |  |
| C*07:01  C*18:01  C*03:03  C*07:02  C*12:03  C*03:04  C*06:02  C*15:02  C*02:02  C*08:02  C*01:02  C*04:01  C*17:01  C*05:01  C*16:01  C*14:02  Missing | 48  2  14  30  15  13  40  7  25  21  4  63  9  16  18  4  55 | 12.50  0.52  3.65  7.81  3.91  3.39  10.42  1.82  6.51  5.47  1.04  16.41  2.34  4.17  4.69  1.04  14.32 | 17  5  3  28  12  17  17  6  11  13  7  36  6  9  10  4  27 | 7.46  2.19  1.32  12.28  5.26  7.46  7.46  2.63  4.82  5.70  3.07  15.79  2.63  3.95  4.39  1.75  11.84 | 0.058  0.085  0.129  0.171  0.2  0.202  0.255  0.481  0.634  0.669  0.688  0.782  0.827  0.885  0.898  0.909  NA | 0.967  0.967  0.967  0.967  0.967  0.967  0.967  0.967  0.967  0.967  0.967  0.967  0.967  0.967  0.967  0.967  NA | 0.52  4.41  0.36  1.56  1.74  1.74  0.67  1.55  0.82  1.19  1.35  0.93  1.14  0.93  1.06  1.09  NA | 0.26  0.90  0.08  0.82  0.74  0.74  0.33  0.43  0.36  0.53  0.31  0.54  0.34  0.35  0.43  0.23  NA | 1.01  31.90  1.20  2.97  4.06  4.12  1.31  5.24  1.80  2.60  6.37  1.59  3.54  2.35  2.50  5.10  NA |
| *HLA-DRB1* |  |  |  |  |  |  |  |  |  |
| DRB1*08:01  DRB1*04:01  DRB1*12:01  DRB1*08:04  DRB1*07:01  DRB1*11:01  DRB1*03:02  DRB1*11:03  DRB1*11:04  DRB1*04:02  DRB1*01:01  DRB1*10:01  DRB1*13:03  DRB1*15:01  DRB1*13:02  DRB1*03:01  DRB1*11:02  DRB1*01:03  DRB1*14:01  DRB1*15:03  DRB1*04:05  DRB1*16:01  DRB1*01:02  DRB1*13:01  Missing | 4  2  9  6  45  22  13  5  5  8  19  3  6  19  19  33  7  4  9  18  8  4  15  24  77 | 1.04  0.52  2.34  1.56  11.72  5.73  3.39  1.30  1.30  2.08  4.95  0.78  1.56  4.95  4.95  8.59  1.82  1.04  2.34  4.69  2.08  1.04  3.91  6.25  20.05 | 7  4  1  2  20  10  3  1  1  2  16  4  2  9  9  18  5  2  5  10  4  2  8  14  69 | 3.07  1.75  0.44  0.88  8.77  4.39  1.32  0.44  0.44  0.88  7.02  1.75  0.88  3.95  3.95  7.89  2.19  0.88  2.19  4.39  1.75  0.88  3.51  6.14  30.26 | 0.078  0.086  0.144  0.249  0.254  0.298  0.356  0.365  0.462  0.487  0.497  0.514  0.548  0.574  0.578  0.645  0.674  0.754  0.754  0.802  0.846  0.857  0.924  0.928  NA | 0.967  0.967  0.967  0.967  0.967  0.967  0.967  0.967  0.967  0.967  0.967  0.967  0.967  0.967  0.967  0.967  0.967  0.967  0.967  0.967  0.967  0.967  0.967  0.967  NA | 3.36  5.35  0.20  0.37  0.69  0.64  0.54  0.36  0.43  0.56  1.31  1.74  0.60  0.77  0.78  1.17  1.32  1.33  0.83  1.12  0.87  0.84  0.95  1.04  NA | 0.88  0.84  0.01  0.05  0.36  0.27  0.12  0.02  0.02  0.08  0.59  0.33  0.09  0.30  0.31  0.59  0.34  0.18  0.23  0.46  0.20  0.10  0.34  0.47  NA | 14.10  45.14  1.20  1.77  1.29  1.44  1.79  2.45  3.04  2.45  2.84  10.10  2.79  1.85  1.83  2.30  4.81  7.29  2.65  2.63  3.27  5.00  2.52  2.21  NA |
| *HLA-DQA1* |  |  |  |  |  |  |  |  |  |
| DQA1*05:01  DQA1*02:01  DQA1*03:01  DQA1*04:01  DQA1*01:01  DQA1*01:03  DQA1*01:02  Missing | 82  49  39  28  52  25  67  42 | 21.35  12.76  10.16  7.29  13.54  6.51  17.45  10.94 | 44  25  30  19  37  12  38  23 | 19.30  10.96  13.16  8.33  16.23  5.26  16.67  10.09 | 0.277  0.347  0.533  0.6  0.604  0.678  0.944  NA | 0.967  0.967  0.967  0.967  0.967  0.967  0.969  NA | 0.75  0.75  1.21  1.20  1.16  0.84  1.02  NA | 0.44  0.40  0.66  0.59  0.66  0.37  0.60  NA | 1.26  1.36  2.19  2.40  2.01  1.85  1.73  NA |
| *HLA-DQB1* |  |  |  |  |  |  |  |  |  |
| DQB1*03:01  DQB1*04:02  DQB1*06:09  DQB1*02:01  DQB1*05:01  DQB1*06:03  DQB1*03:03  DQB1*05:02  DQB1*05:03  DQB1*03:02  DQB1*06:04  DQB1*06:02  Missing | 62  26  7  81  47  26  7  9  9  27  13  40  30 | 16.15  6.77  1.82  21.09  12.24  6.77  1.82  2.34  2.34  7.03  3.39  10.42  7.81 | 34  22  2  40  35  12  7  4  6  18  9  22  17 | 14.91  9.65  0.88  17.54  15.35  5.26  3.07  1.75  2.63  7.89  3.95  9.65  7.46 | 0.165  0.178  0.391  0.394  0.526  0.622  0.788  0.835  0.842  0.856  0.861  0.999  NA | 0.967  0.967  0.967  0.967  0.967  0.967  0.967  0.967  0.967  0.967  0.967  0.999  NA | 0.67  1.60  0.49  0.79  1.20  0.82  1.18  0.87  1.13  1.07  1.09  1.00  NA | 0.38  0.80  0.07  0.47  0.68  0.36  0.34  0.22  0.34  0.52  0.41  0.53  NA | 1.17  3.18  2.18  1.35  2.11  1.79  3.94  3.02  3.50  2.16  2.75  1.86  NA |
| *HLA-DPA1* |  |  |  |  |  |  |  |  |  |
| DPA1*02:02  DPA1*01:03  DPA1*03:03  DPA1*02:01  Missing | 26  105  11  61  181 | 6.77  27.34  2.86  15.89  47.14 | 17  60  7  41  103 | 7.46  26.32  3.07  17.98  45.18 | 0.176  0.5  0.856  0.972  NA | 0.967  0.967  0.967  0.98  NA | 1.65  0.84  1.10  0.99  NA | 0.79  0.50  0.37  0.58  NA | 3.40  1.40  3.08  1.69  NA |
| *HLA-DPB1* |  |  |  |  |  |  |  |  |  |
| DPB1*13:01  DPB1*01:01  DPB1*14:01  DPB1*17:01  DPB1*10:01  DPB1*02:01  DPB1*04:02  DPB1*05:01  DPB1*03:01  DPB1*06:01  DPB1*11:01  DPB1*04:01  Missing | 5  40  9  16  8  53  44  7  26  3  8  79  86 | 1.30  10.42  2.34  4.17  2.08  13.80  11.46  1.82  6.77  0.78  2.08  20.57  22.40 | 9  20  4  7  8  22  36  2  18  3  5  50  44 | 3.95  8.77  1.75  3.07  3.51  9.65  15.79  0.88  7.89  1.32  2.19  21.93  19.30 | 0.041  0.233  0.25  0.325  0.339  0.384  0.523  0.567  0.624  0.674  0.885  0.916  NA | 0.967  0.967  0.967  0.967  0.967  0.967  0.967  0.967  0.967  0.967  0.967  0.967  NA | 3.43  0.67  0.44  0.61  1.71  0.76  1.21  0.61  1.20  1.44  0.91  1.03  NA | 1.08  0.35  0.09  0.22  0.56  0.41  0.67  0.09  0.58  0.24  0.25  0.62  NA | 12.05  1.28  1.65  1.58  5.17  1.39  2.14  2.85  2.42  8.36  3.04  1.72  NA |

Allele frequencies and association analysis between classical HLA alleles and COVID-19 severity under a dominant model.

Sample size: 192 severe cases (384 alleles) and 114 subjects with mild symptoms (228 alleles). Rows labeled as Missing indicate individuals without valid genotype calls, and rows labeled as Rare correspond to the number of alleles excluded from association analysis due to low frequency (< 1% in total cohort). P-values obtained from logistic regression adjusted for age (continuous) and Native American ancestry under dominant model.

Abbreviations: N, number ofalleles; %, frequency; OR, Odds ratio; CI, Confidence Interval; Inf, Inferior; Sup, Superior; FDR: False discovery rates; NA, not available.

**Table S4: Association between COVID-19 severity and amino acid positions at HLA class I and II genes under dominant model.**

| **Amino acid position** | **Residues** | **df** | $x^{2}$ | ***p-value*** | **FDR** |
| --- | --- | --- | --- | --- | --- |
| *HLA-A* |  |  |  |  |  |
| A_127  A_95  A_107  A_74  A_161  A_116  A_66  A_163  A_97  A_171  A_62  A_73  A_114  A_17  A_166  A_167  A_149  A_63  A_65  A_99  A_56  A_142  A_145  A_105  A_70  A_109  A_90  A_43  A_12  A_79  A_80  A_81  A_82  A_83  A_144  A_152  A_76  A_151  A_9  A_77  A_156  A_150  A_158  A_44  A_67 | K, N  L, I, V  G, W  D, H  E, D  Y, D, H  K, N  T, R  M, R, I  Y  E, R, G, Q, L  T, I  H, Q, R, E  R, S  D, E  G, W  A, T  E, N, Q  G, R  F, Y  G, R  I, T  R, H  S, P  H, Q  F, L  A, D  Q, R  V, M  R, G  I, T  A, L  L, R  R, G  K, Q  V, E, A, R, W  E, A, V  H, R  S, Y, F, T  N, S, D  Q, W, L, R  A, V  A, V  R, K  V, M | 2  3  2  2  2  3  2  2  3  1  5  2  4  2  2  2  2  3  2  2  2  2  2  2  2  2  2  2  2  2  2  2  2  2  2  5  3  2  4  3  4  2  2  2  2 | 3.405  4.196  2.475  2.475  2.020  3.048  1.811  1.796  2.890  0.532  4.591  1.391  3.357  1.225  1.117  1.117  1.103  1.816  0.973  0.973  0.960  0.913  0.913  0.909  0.659  0.627  0.577  0.573  0.538  0.375  0.375  0.375  0.375  0.375  0.366  1.688  0.478  0.146  0.863  0.400  0.662  0.029  0.029  0.029  0.029 | 0.182  0.241  0.290  0.290  0.364  0.384  0.404  0.407  0.409  0.466  0.468  0.499  0.500  0.542  0.572  0.572  0.576  0.611  0.615  0.615  0.619  0.633  0.633  0.635  0.719  0.731  0.749  0.751  0.764  0.829  0.829  0.829  0.829  0.829  0.833  0.890  0.924  0.930  0.930  0.940  0.956  0.985  0.985  0.985  0.985 | 0.987  0.987  0.987  0.987  0.987  0.987  0.987  0.987  0.987  0.987  0.987  0.987  0.987  0.987  0.987  0.987  0.987  0.987  0.987  0.987  0.987  0.987  0.987  0.987  0.996  0.996  0.996  0.996  0.996  0.996  0.996  0.996  0.996  0.996  0.996  0.996  0.996  0.996  0.996  0.996  0.996  0.996  0.996  0.996  0.996 |
| *HLA-B* |  |  |  |  |  |
| B_116  B_82  B_83  B_-21  B_-23  B_69  B_71  B_131  B_12  B_143  B_147  B_178  B_81  B_177  B_180  B_80  B_113  B_70  B_74  B_77  B_109  B_145  B_59  B_99  B_245  B_62  B_11  B_41  B_97  B_152  B_-16  B_163  B_282  B_306  B_326  B_65  B_66  B_9  B_63  B_30  B_194  B_158  B_171  B_167  B_46  B_103  B_95  B_67  B_32  B_24  B_156  B_114  B_45  B_94 | Y, S, F, D, L  R, L  G, R  *, T  *, R  T, A  T, A  S, R  M, V  T, S  W, L  T, K  L, A  E, D  Q, E  N, I, T  H, Y  N, S, Q  Y, D  S, N, D  L  R  Y  Y  *, A  R, G  A, S  A, T  R, W, T, S, V  E, V  *, V  L, T, E  *, V  *, A  *, C  Q, R  I, N  Y, H, D  N, E  D, G  *, I  A, T  Y, H  W, S  E, A  V, L  L, I, W  C, F, M, S, Y  Q, L  S, A, T  L, R, D, W  D, H, N  E, T, M, K  T, I | 5  2  2  2  2  2  2  2  2  2  2  2  2  2  2  3  2  3  2  3  1  1  1  1  2  2  2  2  5  2  2  3  2  2  2  2  2  3  2  2  2  2  2  2  2  2  3  5  2  3  4  3  4  2 | 11.184  4.369  4.369  3.602  3.602  3.569  3.569  2.902  2.652  2.595  2.595  2.458  2.357  2.264  2.264  3.404  2.101  3.183  1.979  3.093  0.775  0.775  0.775  0.775  1.924  1.913  1.911  1.864  5.120  1.749  1.556  2.570  1.366  1.366  1.366  1.253  1.253  2.138  1.166  1.021  1.008  0.914  0.896  0.854  0.851  0.795  1.347  2.534  0.510  0.709  1.155  0.596  0.736  0.028 | 0.048  0.113  0.113  0.165  0.165  0.168  0.168  0.234  0.266  0.273  0.273  0.293  0.308  0.322  0.322  0.333  0.350  0.364  0.372  0.377  0.379  0.379  0.379  0.379  0.382  0.384  0.385  0.394  0.401  0.417  0.459  0.463  0.505  0.505  0.505  0.534  0.534  0.544  0.558  0.600  0.604  0.633  0.639  0.652  0.653  0.672  0.718  0.771  0.775  0.871  0.885  0.897  0.947  0.986 | 0.987  0.987  0.987  0.987  0.987  0.987  0.987  0.987  0.987  0.987  0.987  0.987  0.987  0.987  0.987  0.987  0.987  0.987  0.987  0.987  0.987  0.987  0.987  0.987  0.987  0.987  0.987  0.987  0.987  0.987  0.987  0.987  0.987  0.987  0.987  0.987  0.987  0.987  0.987  0.987  0.987  0.987  0.987  0.987  0.987  0.991  0.996  0.996  0.996  0.996  0.996  0.996  0.996  0.996 |
| *HLA-C* |  |  |  |  |  |
| C_66  C_91  C_73  C_90  C_99  C_77  C_80  C_152  C_6  C_14  C_49  C_177  C_11  C_16  C_138  C_35  C_114  C_147  C_113  C_103  C_173  C_143  C_170  C_21  C_156  C_94  C_95  C_116  C_163  C_97  C_9  C_24 | K, N  G, R  T, A  A, D  Y, F, S  S, N  N, K  E, A  R  R, W  A, E  E, K  A, S  G, S  T, K  R, Q  D, N  W, L  Y, H  V, L  K, E  T, S  R, G  H, R  L, R, W, Q  I, T  I, L  Y, F, S, L  L, T, E  R, W  Y, S, D  A, S | 2  2  2  2  3  2  2  2  1  2  2  2  2  2  2  2  2  2  2  2  2  2  2  2  4  2  2  4  3  2  3  2 | 2.892  2.838  2.043  1.889  2.031  1.121  1.121  0.909  0.197  0.723  0.723  0.672  0.653  0.592  0.575  0.575  0.459  0.392  0.370  0.360  0.360  0.255  0.255  0.234  1.115  0.145  0.134  0.811  0.290  0.056  0.158  0.009 | 0.236  0.242  0.360  0.389  0.566  0.571  0.571  0.635  0.657  0.697  0.697  0.715  0.721  0.744  0.750  0.750  0.795  0.822  0.831  0.835  0.835  0.880  0.880  0.890  0.892  0.930  0.935  0.937  0.962  0.972  0.984  0.996 | 0.987  0.987  0.987  0.987  0.987  0.987  0.987  0.987  0.987  0.996  0.996  0.996  0.996  0.996  0.996  0.996  0.996  0.996  0.996  0.996  0.996  0.996  0.996  0.996  0.996  0.996  0.996  0.996  0.996  0.996  0.996  0.996 |
| *HLA-DRB1* |  |  |  |  |  |
| DRB1_70  DRB1_38  DRB1_40  DRB1_33  DRB1_85  DRB1_32  DRB1_31  DRB1_9  DRB1_10  DRB1_25  DRB1_12  DRB1_78  DRB1_47  DRB1_16  DRB1_67  DRB1_11  DRB1_57  DRB1_60  DRB1_77  DRB1_14  DRB1_58  DRB1_13  DRB1_28  DRB1_73  DRB1_86  DRB1_30  DRB1_37  DRB1_26  DRB1_71  DRB1_74 | Q, D, R  V  F  N, H  V, A  H, Y  F, I  E, W  Y, Q  R, Q  T, K  Y, V  F, Y  H, Y  L, I, F  S, V, L, G, P  D, A, V, S  Y, H, S  N, T  E, K  A, E  S, H, F, Y, G, R  D, E  G, A  V, G  Y, C, L, H  N, Y, F, S  Y, F, L  K, E, R, A  R, A, E, Q, L | 3  1  1  2  2  2  2  2  2  2  2  2  2  2  3  5  4  3  2  2  2  6  2  2  2  4  4  3  4  5 | 7.256  3.067  3.067  4.563  3.900  3.890  3.809  3.567  3.163  2.862  2.828  2.664  2.606  2.515  3.669  5.835  4.665  3.430  2.182  2.127  1.981  6.345  1.879  1.856  1.841  3.880  2.566  1.578  2.180  2.207 | 0.064  0.080  0.080  0.102  0.142  0.143  0.149  0.168  0.206  0.239  0.243  0.264  0.272  0.284  0.299  0.323  0.323  0.330  0.336  0.345  0.371  0.386  0.391  0.395  0.398  0.422  0.633  0.664  0.703  0.820 | 0.987  0.987  0.987  0.987  0.987  0.987  0.987  0.987  0.987  0.987  0.987  0.987  0.987  0.987  0.987  0.987  0.987  0.987  0.987  0.987  0.987  0.987  0.987  0.987  0.987  0.987  0.987  0.991  0.996  0.996 |
| *HLA-DQA1* |  |  |  |  |  |
| DQA1_130  DQA1_11  DQA1_25  DQA1_69  DQA1_41  DQA1_18  DQA1_45  DQA1_48  DQA1_55  DQA1_61  DQA1_64  DQA1_66  DQA1_80  DQA1_52  DQA1_75  DQA1_175  DQA1_54  DQA1_207  DQA1_56  DQA1_76  DQA1_218  DQA1_26  DQA1_34  DQA1_129  DQA1_47  DQA1_40  DQA1_51  DQA1_50  DQA1_53 | *, S, A  Y, C  Y, F  L, A, T  R, K  S, F  V, A  L, W  R, G  F, G  T, R  I, M  S, Y  R, S, H  S, I  *, Q, E  F, L  *, V, M  ., G, R  L, M, V  *, Q, R  T, S  Q, E  *, Q, H  C, R, Q, K  G, E  L, F  V, E, L  Q, K, R | 3  2  2  3  2  2  2  2  2  2  2  2  2  3  2  3  2  3  3  3  3  2  2  3  4  2  2  3  3 | 4.359  2.712  2.390  2.727  1.505  1.496  1.496  1.496  1.496  1.496  1.496  1.496  1.496  2.355  1.199  1.840  0.992  1.732  1.647  1.647  1.563  0.784  0.743  1.399  1.968  0.221  0.221  0.332  0.332 | 0.225  0.258  0.303  0.436  0.471  0.473  0.473  0.473  0.473  0.473  0.473  0.473  0.473  0.502  0.549  0.606  0.609  0.630  0.649  0.649  0.668  0.676  0.690  0.706  0.742  0.895  0.895  0.954  0.954 | 0.987  0.987  0.987  0.987  0.987  0.987  0.987  0.987  0.987  0.987  0.987  0.987  0.987  0.987  0.987  0.987  0.987  0.987  0.987  0.987  0.991  0.991  0.996  0.996  0.996  0.996  0.996  0.996  0.996 |
| *HLA-DQB1* |  |  |  |  |  |
| DQB1_9  DQB1_66  DQB1_67  DQB1_53  DQB1_45  DQB1_56  DQB1_14  DQB1_70  DQB1_84  DQB1_85  DQB1_89  DQB1_90  DQB1_26  DQB1_86  DQB1_55  DQB1_87  DQB1_74  DQB1_13  DQB1_30  DQB1_28  DQB1_37  DQB1_46  DQB1_47  DQB1_52  DQB1_71  DQB1_75  DQB1_38  DQB1_57  DQB1_77 | Y, *, F  D, E  I, V  L, Q  G, E  P, L  M, *, L  R, *, E, G  Q, *, E  L, *, V  T, *, G  T, *, I  L, G, Y  E, *, A, G  L, P, R  L, *, F, Y  A, E, *, S  G, *, A  S, Y, H  S, T  I, Y  E, V  F, Y  L, P  K, T, *, D, A  V, L, *  V, A  A, D, S, V  R, T, * | 3  2  2  2  2  2  3  4  3  3  3  3  3  4  3  4  4  3  3  2  2  2  2  2  5  3  2  4  3 | 4.745  3.222  3.222  2.819  2.375  2.286  2.883  3.782  2.660  2.660  2.660  2.660  2.579  3.330  2.292  3.240  3.149  2.150  2.055  1.152  1.152  1.152  1.152  1.152  3.615  1.718  0.067  0.457  0.072 | 0.191  0.200  0.200  0.244  0.305  0.319  0.410  0.436  0.447  0.447  0.447  0.447  0.461  0.504  0.514  0.519  0.533  0.542  0.561  0.562  0.562  0.562  0.562  0.562  0.606  0.633  0.967  0.978  0.995 | 0.987  0.987  0.987  0.987  0.987  0.987  0.987  0.987  0.987  0.987  0.987  0.987  0.987  0.987  0.987  0.987  0.987  0.987  0.987  0.987  0.987  0.987  0.987  0.987  0.987  0.987  0.996  0.996  0.996 |
| HLA-DPA1 |  |  |  |  |  |
| DPA1_11  DPA1_50  DPA1_83  DPA1_31  DPA1_18  DPA1_72  DPA1_73  DPA1_28  DPA1_66 | A, M  R, Q  A, T  Q, M  P  T  L  E, D  L, S | 2  2  2  2  1  1  1  2  2 | 1.554  0.441  0.441  0.436  0.005  0.005  0.005  0.114  0.044 | 0.460  0.802  0.802  0.804  0.942  0.942  0.942  0.945  0.978 | 0.987  0.996  0.996  0.996  0.996  0.996  0.996  0.996  0.996 |
| *HLA-DPB1* |  |  |  |  |  |
| DPB1_76  DPB1_215  DPB1_11  DPB1_170  DPB1_96  DPB1_57  DPB1_205  DPB1_85  DPB1_86  DPB1_87  DPB1_65  DPB1_33  DPB1_8  DPB1_84  DPB1_9  DPB1_69  DPB1_56  DPB1_35  DPB1_36  DPB1_55 | V, M, I  *, I  G, L  *, I, T  *, K, R  E, D  *, V  E, G  A, P  V, M  I, L  E, Q  V, L  D, G  Y, F, H  K, E, R  A, E  Y, F, L  A, V  A, D, E | 3  2  2  3  3  2  2  2  2  2  2  2  2  2  3  3  2  3  2  3 | 5.622  2.786  2.731  3.911  3.911  2.272  2.268  2.248  2.248  2.248  1.617  1.258  1.257  0.760  1.044  0.788  0.058  0.210  0.031  0.105 | 0.132  0.248  0.255  0.271  0.271  0.321  0.322  0.325  0.325  0.325  0.445  0.533  0.533  0.684  0.791  0.852  0.971  0.976  0.984  0.991 | 0.987  0.987  0.987  0.987  0.987  0.987  0.987  0.987  0.987  0.987  0.987  0.987  0.987  0.996  0.996  0.996  0.996  0.996  0.996  0.996 |

Abbreviations: df, Degrees of freedom; x², Likelihood ratio; FDR: False discovery rates. P-values obtained from logistic regression adjusted for age (continuous) and Native American ancestry.

**Table S5: Association between each amino acid at HLA-B 116 site COVID-19 severity and under dominant model.**

| **Amino acid** | **Mild symptoms** | | **Severe disease** | |  |  |  | **_95%_CI** | |
| --- | --- | --- | --- | --- | --- | --- | --- | --- | --- |
| **position/residue** | **N** | % | **N** | % | ***p-value*** | **FDR** | **OR** | **Inf** | **Sup** |
| B*116_S  B*116_F  B*116_L  B*116_Y  B*116_D  Missing | 90  35  38  88  41  92 | 23.44  9.11  9.89  22.92  10.68  23.96 | 36  27  17  50  23  75 | 15.79  11.84  7.46  21.93  10.09  32.89 | 0.006  0.090  0.685  0.914  0.980  NA | 0.031  0.225  0.980  0.980  0.980  NA | 0.48  1.70  0.87  1.03  0.99  NA | 0.28  0.92  0.43  0.62  0.53  NA | 0.81  3.13  1.70  1.72  1.83  NA |

Results were obtained through logistic regression analysis collapsing all HLA alleles encoding the same residue. Rows labeled as Missing indicate individuals without valid genotype calls. P-values obtained from logistic regression adjusted for age (continuous) and Native American ancestry under dominant model.

Abbreviations: N, number ofalleles; %, frequency; OR, Odds ratio; CI, Confidence Interval; Inf, Inferior; Sup, Superior; FDR: False discovery rates; NA, not available.

**Table S6: Alleles carrying each amino acid residue at HLA-B 116 site.**

| **Amino acid**  **position 116** | **Allele** | **N** | **%** |
| --- | --- | --- | --- |
| D | *15:17, *27:03, *27:05, *44:02, *44:03 | 72 | 12.04 |
| F | *14:01, *14:02, *35:03, *37:01, *38:01, *39:06 | 65 | 10.87 |
| L | *13:02, *45:01, *49:01, *50:01, *55:01 | 59 | 9.87 |
| S | *15:01, *15:03, *18:01, *35:01, *35:08, *53:01, *57:01, *58:01, *58:02 | 149 | 24.92 |
| Y | *07:02, *08:01, *15:10, *35:02, *40:01, *40:02, *40:04, *41:01, *42:01, *51:01, *52:01, *57:03, *81:01 | 167 | 27.93 |

Abbreviations: N, number of participants; %, frequency.

**Table S7: Strong-binding peptides from the SARS-CoV-2 Spike protein (Wuhan strain) to HLA-B allotypes carrying serine at position 116.**

| **Peptide** | **HLA allotypes** **Binding** |
| --- | --- |
| VASQSIIAY | HLA-B*15:01, HLA-B*15:03, HLA-B*35:01, HLA-B*53:01, HLA-B*58:01 |
| QSAPHGVVF | HLA-B*15:01, HLA-B*15:03, HLA-B*35:01, HLA-B*58:01 |
| FAMQMAYRF | HLA-B*35:01, HLA-B*53:01, HLA-B*58:01 |
| HADQLTPTW | HLA-B*35:01, HLA-B*53:01, HLA-B*58:01 |
| IPFAMQMAY | HLA-B*18:01, HLA-B*35:01, HLA-B*53:01 |
| LPFFSNVTW | HLA-B*35:01, HLA-B*53:01, HLA-B*58:01 |
| NQKLIANQF | HLA-B*15:01, HLA-B*15:03, HLA-B*18:01 |
| FPQSAPHGV | HLA-B*35:01, HLA-B*53:01 |
| FQPTNGVGY | HLA-B*15:01, HLA-B*15:03 |
| FVFKNIDGY | HLA-B*15:01, HLA-B*35:01 |
| GQTGKIADY | HLA-B*15:01, HLA-B*15:03 |
| GVYYPDKVF | HLA-B*15:01, HLA-B*15:03 |
| IPTNFTISV | HLA-B*35:01, HLA-B*53:01 |
| LAGTITSGW | HLA-B*53:01, HLA-B*58:01 |
| LPFNDGVYF | HLA-B*35:01, HLA-B*53:01 |
| LPPAYTNSF | HLA-B*35:01, HLA-B*53:01 |
| LPPLLTDEM | HLA-B*35:01, HLA-B*53:01 |
| LQIPFAMQM | HLA-B*15:01, HLA-B*15:03 |
| NSIAIPTNF | HLA-B*53:01, HLA-B*58:01 |
| QLTPTWRVY | HLA-B*15:01, HLA-B*15:03 |
| QPRTFLLKY | HLA-B*35:01, HLA-B*53:01 |
| QPTESIVRF | HLA-B*35:01, HLA-B*53:01 |
| SQSIIAYTM | HLA-B*15:01, HLA-B*15:03 |
| TLLALHRSY | HLA-B*15:01, HLA-B*15:03 |
| TPCNGVEGF | HLA-B*35:01, HLA-B*53:01 |
| TSNQVAVLY | HLA-B*35:01, HLA-B*58:01 |
| VLKGVKLHY | HLA-B*15:01, HLA-B*15:03 |
| AHFPREGVF | HLA-B*15:03 |
| AQKFNGLTV | HLA-B*15:03 |
| ASFSTFKCY | HLA-B*15:01 |
| CVADYSVLY | HLA-B*35:01 |
| DAVRDPQTL | HLA-B*53:01 |
| DEDDSEPVL | HLA-B*18:01 |
| DEMIAQYTS | HLA-B*18:01 |
| FERDISTEI | HLA-B*18:01 |
| FEYVSQPFL | HLA-B*18:01 |
| FKNLREFVF | HLA-B*15:03 |
| GEVFNATRF | HLA-B*18:01 |
| GKGYHLMSF | HLA-B*15:03 |
| GRLQSLQTY | HLA-B*15:03 |
| GTITSGWTF | HLA-B*58:01 |
| GVVFLHVTY | HLA-B*15:01 |
| IAIPTNFTI | HLA-B*58:01 |
| KKFLPFQQF | HLA-B*15:03 |
| LGAENSVAY | HLA-B*35:01 |
| LQTYVTQQL | HLA-B*15:03 |
| LTDEMIAQY | HLA-B*35:01 |
| LVKQLSSNF | HLA-B*15:01 |
| LVRDLPQGF | HLA-B*15:01 |
| NATRFASVY | HLA-B*35:01 |
| NCYFPLQSY | HLA-B*35:01 |
| NDLCFTNVY | HLA-B*18:01 |
| NSFTRGVYY | HLA-B*35:01 |
| QELGKYEQY | HLA-B*18:01 |
| QIITTDNTF | HLA-B*15:01 |
| QKFNGLTVL | HLA-B*15:03 |
| RSFIEDLLF | HLA-B*58:01 |
| SANNCTFEY | HLA-B*35:01 |
| SEFRVYSSA | HLA-B*18:01 |
| SKVGGNYNY | HLA-B*15:03 |
| TEVPVAIHA | HLA-B*18:01 |
| TPGDSSSGW | HLA-B*53:01 |
| TPINLVRDL | HLA-B*53:01 |
| TRTQLPPAY | HLA-B*15:03 |
| VGYLQPRTF | HLA-B*15:03 |
| VLHSTQDLF | HLA-B*15:01 |
| VRFPNITNL | HLA-B*15:03 |
| WMESEFRVY | HLA-B*15:01 |
| YEQYIKWPW | HLA-B*18:01 |
| YKTPPIKDF | HLA-B*15:03 |
| YNSASFSTF | HLA-B*15:03 |
| YSSANNCTF | HLA-B*58:01 |

**Table S8: Strong-binding peptides from the SARS-CoV-2 Spike protein (Omicron BA.2.12.1 variant) to HLA-B allotypes carrying serine at position 116.**

| **Peptide** | **HLA allotypes Binding** |
| --- | --- |
| VASQSIIAY | HLA-B*15:01, HLA-B*15:03, HLA-B*35:01, HLA-B*53:01, HLA-B*58:01 |
| QSAPHGVVF | HLA-B*15:01, HLA-B*15:03, HLA-B*35:01, HLA-B*58:01 |
| HADQLTPTW | HLA-B*35:01, HLA-B*53:01, HLA-B*58:01 |
| IPFPMQMAY | HLA-B*15:03, HLA-B*35:01, HLA-B*53:01 |
| LPFFSNVTW | HLA-B*35:01, HLA-B*53:01, HLA-B*58:01 |
| NLITRTQSY | HLA-B*15:01, HLA-B*15:03, HLA-B*35:01 |
| RSYGFRPTY | HLA-B*15:01, HLA-B*15:03, HLA-B*58:01 |
| RTQSYTNSF | HLA-B*15:01, HLA-B*15:03, HLA-B*58:01 |
| FPMQMAYRF | HLA-B*35:01, HLA-B*53:01 |
| FPQSAPHGV | HLA-B*35:01, HLA-B*53:01 |
| FVFKNIDGY | HLA-B*15:01, HLA-B*35:01 |
| GQTGNIADY | HLA-B*15:01, HLA-B*15:03 |
| GVYYPDKVF | HLA-B*15:01, HLA-B*15:03 |
| IPTNFTISV | HLA-B*35:01, HLA-B*53:01 |
| LAGTITSGW | HLA-B*53:01, HLA-B*58:01 |
| LPFNDGVYF | HLA-B*35:01, HLA-B*53:01 |
| LPPLLTDEM | HLA-B*35:01, HLA-B*53:01 |
| LQIPFPMQM | HLA-B*15:01, HLA-B*15:03 |
| NQKLIANQF | HLA-B*15:01, HLA-B*15:03 |
| NSIAIPTNF | HLA-B*53:01, HLA-B*58:01 |
| QLTPTWRVY | HLA-B*15:01, HLA-B*15:03 |
| QPRTFLLKY | HLA-B*35:01, HLA-B*53:01 |
| QPTESIVRF | HLA-B*35:01, HLA-B*53:01 |
| SQSIIAYTM | HLA-B*15:01, HLA-B*15:03 |
| TLLALHRSY | HLA-B*15:01, HLA-B*15:03 |
| TSNQVAVLY | HLA-B*35:01, HLA-B*58:01 |
| VLKGVKLHY | HLA-B*15:01, HLA-B*15:03 |
| AHFPREGVF | HLA-B*15:03 |
| APFFAFKCY | HLA-B*35:01 |
| AQKFNGLTV | HLA-B*15:03 |
| CASYQTQTY | HLA-B*35:01 |
| CVADYSVLY | HLA-B*35:01 |
| DAVRDPQTL | HLA-B*53:01 |
| FKNLREFVF | HLA-B*15:03 |
| GKGYHLMSF | HLA-B*15:03 |
| GRLQSLQTY | HLA-B*15:03 |
| GTITSGWTF | HLA-B*58:01 |
| GVVFLHVTY | HLA-B*15:01 |
| IAIPTNFTI | HLA-B*58:01 |
| IKYFGGFNF | HLA-B*15:03 |
| KKFLPFQQF | HLA-B*15:03 |
| LGAENLVAY | HLA-B*35:01 |
| LQTYVTQQL | HLA-B*15:03 |
| LTDEMIAQY | HLA-B*35:01 |
| LVKQLSSKF | HLA-B*15:01 |
| NATRFASVY | HLA-B*35:01 |
| NSFTRGVYY | HLA-B*35:01 |
| QIITTDNTF | HLA-B*15:01 |
| QKFNGLTVL | HLA-B*15:03 |
| SANNCTFEY | HLA-B*35:01 |
| SKVGGNYNY | HLA-B*15:03 |
| TPGDSSSGW | HLA-B*53:01 |
| VGYLQPRTF | HLA-B*15:03 |
| VLHSTQDLF | HLA-B*15:01 |
| VRFPNITNL | HLA-B*15:03 |
| WMESEFRVY | HLA-B*15:01 |
| YKTPPIKYF | HLA-B*15:03 |
| YSSANNCTF | HLA-B*58:01 |

**Figure S2: *Manhattan plot* showing association analyses between single nucleotide variants at HLA region and COVID-19 severity.**


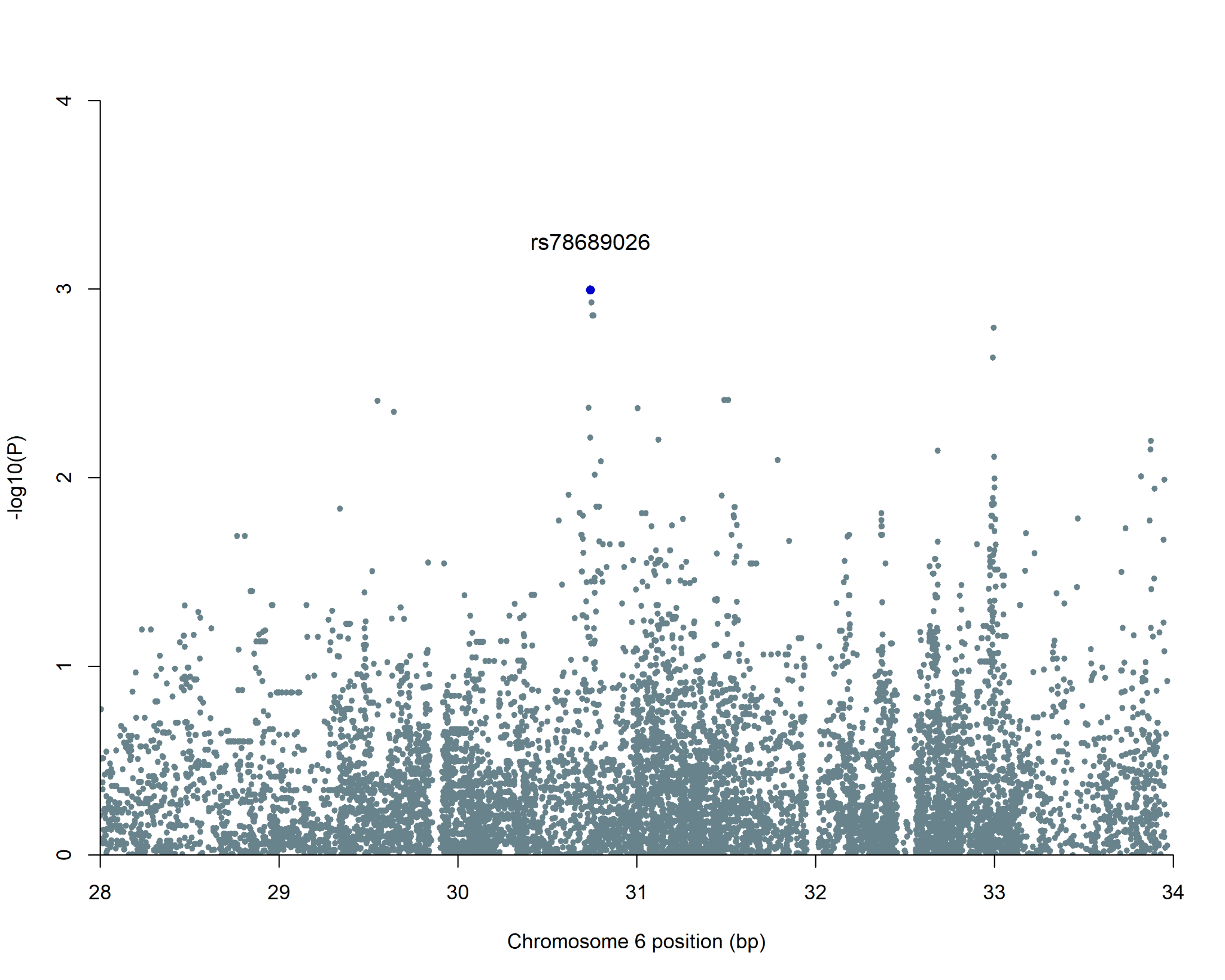


P-values were obtained through logistic regression under an additive model adjusted for age and Native American ancestry. The dark blue point represents the lead SNV with lowest p-value (rs78689026; NC_000006.11:g.30740038G>A; *HCG20*, intronic; OR = 3.05; _95%_CI = 1.57 - 5.92; p = 0.001; _adjusted_ p = 0.43).
